# Supplementary material for: Role of physiotherapy in supporting recovery from breast cancer treatment: a qualitative study embedded within the UK PROSPER trial
Source: BMJ Open. 2021 May 11;11(5):e040116. doi: 10.1136/bmjopen-2020-040116 (PMC8118023; doi:10.1136/bmjopen-2020-040116)
Supplement: Author's manuscript [file bmjopen-2020-040116.draft_revisions.pdf]

BMJ Open is committed to open peer review. As part of this commitment we make the peer review history of every article we publish publicly available.

When an article is published we post the peer reviewers' comments and the authors' responses online. We also post the versions of the paper that were used during peer review. These are the versions that the peer review comments apply to.

The versions of the paper that follow are the versions that were submitted during the peer review process. They are not the versions of record or the final published versions. They should not be cited or distributed as the published version of this manuscript.

BMJ Open is an open access journal and the full, final, typeset and author-corrected version of record of the manuscript is available on our site with no access controls, subscription charges or pay-per-view fees (http://bmjopen.bmj.com).

If you have any questions on BMJ Open's open peer review process please email info.bmjopen@bmj.com

BMJ Open

The role of physiotherapy in supporting recovery from breast cancer treatment: A qualitative study embedded within the UK PROSPER trial

Journal:	*BMJ Open*
Manuscript ID	bmjopen-2020-040116
Article Type:	Original research
Date Submitted by the Author:	06-May-2020
Complete List of Authors:	Rees, Sophie; University of Warwick, Warwick Clinical Trials Unit<br>Mazuquin, Bruno; University of Warwick, Warwick Clinical Trials Unit<br>Richmond, Helen ; University of Warwick, Warwick Clinical Trials Unit; Memorial University of Newfoundland, Faculty of Medicine<br>Williamson, Esther; Nuffield Department of Orthopaedics, Rheumatology and Musculoskeletal Sciences, University of Oxford, United Kingdom. , Centre for Rehabilitation Research in Oxford<br>Bruce, Julie; University of Warwick, Warwick Clinical Trials Unit<br>-, UK PROSPER Study Group; University of Warwick, Warwick Clinical Trials Unit
Keywords:	QUALITATIVE RESEARCH, Breast surgery < SURGERY, PREVENTIVE MEDICINE

SCHOLARONE™
Manuscripts

I, the Submitting Author has the right to grant and does grant on behalf of all authors of the Work (as defined in the below author licence), an exclusive licence and/or a non-exclusive licence for contributions from authors who are: i) UK Crown employees; ii) where BMJ has agreed a CC-BY licence shall apply, and/or iii) in accordance with the terms applicable for US Federal Government officers or employees acting as part of their official duties; on a worldwide, perpetual, irrevocable, royalty-free basis to BMJ Publishing Group Ltd ("BMJ") its licensees and where the relevant Journal is co-owned by BMJ to the co-owners of the Journal, to publish the Work in this journal and any other BMJ products and to exploit all rights, as set out in our licence.

The Submitting Author accepts and understands that any supply made under these terms is made by BMJ to the Submitting Author unless you are acting as an employee on behalf of your employer or a postgraduate student of an affiliated institution which is paying any applicable article publishing charge ("APC") for Open Access articles. Where the Submitting Author wishes to make the Work available on an Open Access basis (and intends to pay the relevant APC), the terms of reuse of such Open Access shall be governed by a Creative Commons licence – details of these licences and which Creative Commons licence will apply to this Work are set out in our licence referred to above.

Other than as permitted in any relevant BMJ Author's Self Archiving Policies, I confirm this Work has not been accepted for publication elsewhere, is not being considered for publication elsewhere and does not duplicate material already published. I confirm all authors consent to publication of this Work and authorise the granting of this licence.

For peer review only

The role of physiotherapy in supporting recovery from breast cancer treatment: A qualitative study embedded within the UK PROSPER trial

Rees S,[1] Mazuquin B,[1] Richmond H,[1,2] Williamson E,[3] Bruce J.[1] on behalf of the UK PROSPER Study Group[1]

[1]*Warwick Clinical Trials Unit, University of Warwick Medical School, Coventry, UK CV4 7AL*

[2]*Faculty of Medicine, Memorial University of Newfoundland, St John's, Canada*

[3]*The Centre for Rehabilitation Research, Nuffield Department of Orthopaedics, Rheumatology and Musculoskeletal Sciences, University of Oxford*

Corresponding author:
Dr Sophie Rees
S.Rees.1@warwick.ac.uk
02476 151 135

For peer review only

Abstract

Objectives: To explore breast cancer patients' experience of taking part in an early physiotherapy-led exercise intervention compared with the experiences of those receiving usual care. To understand physiotherapists' experience of delivering the trial intervention. To explore future strategies for implementation of the intervention from participant and physiotherapist perspective

Design: Qualitative audio-recorded semi-structured interviews with thematic analysis.

Setting: UK National Health Service (NHS)

Participants: Twenty participants at high risk of shoulder problems after breast cancer surgery, recruited to the UK Prevention of Shoulder Problems (PROSPER) Trial (ten each from the intervention arm and control arm respectively); and eleven physiotherapists who delivered the intervention.

Results: Participants described that the PROSPER exercise intervention helped them feel confident in what their body could do, and helped them regain a sense of control in the context of cancer treatment which was largely disempowering. Control arm participants expressed less of a sense of control over their wellbeing. Physiotherapists found the exercise intervention enjoyable to deliver and felt it was valuable to their patients. The extra time allocated to patients during intervention delivery made physiotherapists feel they were providing

For peer review only - http://bmjopen.bmj.com/site/about/guidelines.xhtml

optimal care, being the 'perfect physio'. Lessons were learned about the implementation of a complex exercise intervention for breast cancer patients.

Conclusions: A physiotherapist-delivered early supported exercise intervention with integrated behavioural strategies helped women at risk of shoulder problems following breast cancer treatment to regain control and feel more confident in their ability to mobilise their arm post-surgery. A physiotherapist-delivered early supported exercise intervention with integrated behavioural strategies may address the sense of powerlessness that many patients experience during cancer treatment.

Keywords: *breast cancer; physiotherapy; rehabilitation; prevention; shoulder; qualitative research*

Acknowledgements

This study was embedded within a multicentre UK randomised controlled trial (ISCRTN35358984) and was funded by the National Institute for Health Research Health Technology Assessment Programme (HTA 13/84/10). The research team would like to thank the trial participants and physiotherapists who participated in the qualitative study.

Word count: 3,295

For peer review only

Article Summary

Strengths and limitations of this study

- Interviewing multiple groups (intervention arm, control arm participants, and physiotherapists) in this study enabled us to triangulate the data and explore experiences from multiple perspectives.
- We note that the participants we interviewed were a particularly motivated group, and it is possible we did not capture some of the challenges which other, less motivated, women may have experienced.
- We obtained consent to be approached for interview prior to randomisation, independent of treatment allocation, in an attempt to minimise bias
- We used a convenience sampling approach, which is a potential weakness of this study as it may have resulted in a lack of diversity amongst participants.
- Our sample was overwhelmingly white, with only one of the participants identifying as another ethnic identity.

Introduction

Treatment to the chest and axilla for breast cancer can result in upper body problems, such as reduced range of movement in the shoulder, muscle weakness, pain, lymphoedema, and functional limitations [1, 2]. These problems can impact on ability to carry out activities of daily living, and may persist for many years after treatment [1, 2]. Exercise in the acute phase following breast cancer surgery may improve shoulder function in women at high risk of shoulder problems [1]. Guidelines state that breast cancer patients should be referred to physiotherapy when indicated [3, 4], however, in the UK NHS this is not routine practice. There is a need for a proactive model of care which encourages early exercise-based rehabilitation and provides physiotherapists with resources to inform their practice [5]. Loss of a sense of control, loss of self-identity, and alienation from their bodies during and after treatment are often reported by breast cancer patients [6-12]. It has been proposed that improving women's self-efficacy through physical rehabilitation may improve their quality of life [6].

The UK Prevention Of Shoulder Problems (PROSPER) Trial evaluated the clinical and cost-effectiveness of an early supported home-based physiotherapist-led exercise intervention in women with newly diagnosed breast

For peer review only - http://bmjopen.bmj.com/site/about/guidelines.xhtml

For peer review only

cancer at higher risk of developing shoulder problems after treatment [13, 14]. A description of the intervention and trial protocol have been published [14]. This paper reports the findings of the UK PROSPER trial embedded qualitative study. Figure 1 illustrates the pathway of participants through the trial and embedded qualitative study.

The aims of the qualitative study were:

- To understand the acceptability of the exercise intervention to participants
- To explore how the exercise intervention or control affected their experiences of recovery after cancer treatment.
- To investigate the experiences of physiotherapists delivering the exercise intervention.
- To explore future strategies for implementation of the intervention in the UK NHS setting from the participant and physiotherapist perspective.

For peer review only

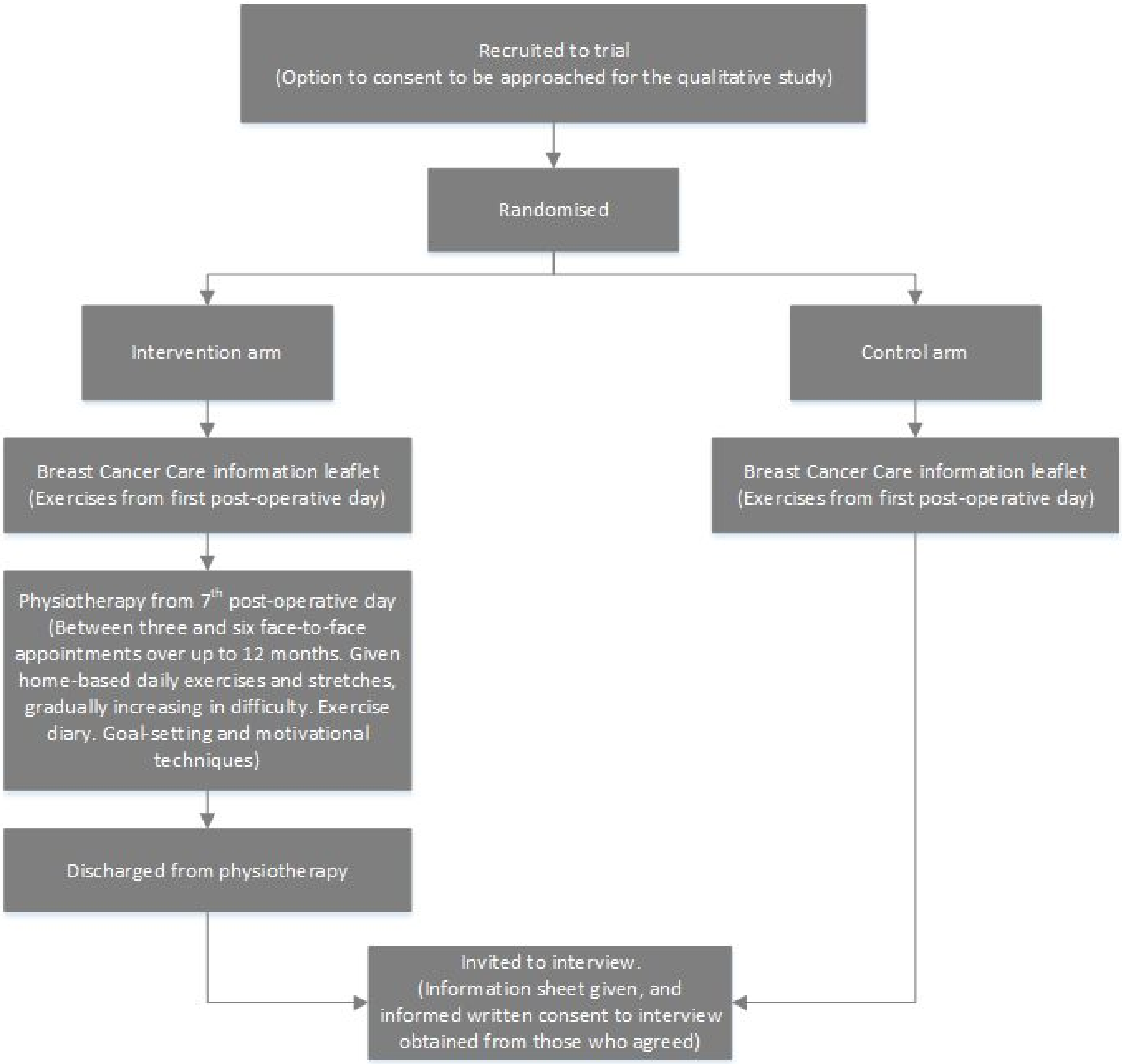

Figure 1 - Participant pathway through trial and qualitative study

only

Methods

Methodology

The study was underpinned by critical realism, assuming that an underlying reality is experienced and given meaning by individuals [15, 16]. To meet the study aims, we conducted qualitative semi-structured interviews with reflexive thematic analysis [17]. This allowed for exploration, depth, and understanding of the experiences of trial participants, thus taking an interpretive 'sense-making' approach rather than hypothesis-testing or confirmatory approach. We used the SRQR reporting guidelines checklist [18].

For peer review only - http://bmjopen.bmj.com/site/about/guidelines.xhtml

Sampling and recruitment

Trial participant interviews

On recruitment to the trial, we offered all trial participants the option to take part in an interview at a later date (see Figure 1). We recorded signed consent to be approached for interview and this formed our sampling frame for the qualitative study. Women in the intervention arm were approached after they were discharged from physiotherapy to avoid contamination bias. The researcher (SR) telephoned participants to invite them to interview, and, if they expressed an interest, participants were sent an information sheet and interview consent form.

After conducting and analysing seven interviews with intervention participants, we decided to interview control arm participants, to compare their experiences. We used our database of those who had consented pre-randomisation to select a sample comparable to the intervention sample in terms of time since randomisation, so that women were at similar stages of postoperative treatment and could reflect back over their experiences of recovery.

Physiotherapist interviews

All physiotherapists delivering the intervention were informed about the interview study. We then sampled physiotherapists from low and high recruiting trial sites to allow exploration of different perspectives on intervention delivery. Therapists were approached by the researcher via email or telephone.

Data collection

Flexible topic guides were developed by the research team and with breast cancer patients, based on the aims of the study and relevant literature. One-off interviews were conducted either at the participant's home, by telephone (trial participants), or in a private room at their place of work (physiotherapists). Physiotherapists who worked together were interviewed in pairs. Only the researcher and interviewees were present. All interviews were audio-recorded. Informed consent was gained before the interview began. The study was approved by the National Research Ethics Service Committee West Midlands Solihull on 20th July 2015 (Ref no. 15/WM/0224),

For peer review only

Data analysis

Interviews were transcribed verbatim, checked for accuracy and anonymity by the researcher (SR), and then uploaded to QSR NVivo Pro 11 [19]. Thematic analysis [20, 21] was conducted by SR. Analysis began alongside data collection. The research team met regularly to discuss emerging findings and the evolving analysis [22]. Saturation in this study meant that we had enough data to understand each of the identified categories and themes, rather than that there was 'nothing new' to be found [23, 24]. We reached saturation after fifteen trial participant interviews, and five physiotherapist interviews.

Reflexivity and rigour

Interviews were conducted sensitively by a female researcher experienced in interviewing cancer patients (SR) [12, 25-28]. The evolving analysis was discussed with the research team (SR/JB/HR/BM). SR is a social scientist with expertise in qualitative research with people with health conditions, including breast cancer, and healthcare professionals. HR and BM are researchers and physiotherapists. JB is a trialist and PROSPER Chief Investigator, she did not influence the qualitative study findings, but provided important contextual details regarding the trial and intervention. We were careful to conduct balanced interviews, without assuming that the trial participants and physiotherapists would have positive views of the intervention. SR reminded interviewees throughout that she was not involved in the development of the intervention, and welcomed their honest views. Rigour was assessed using Lincoln and Guba's conceptualisation of trustworthiness [29]. SR collected the data and was immersed in the data during analysis. Quotes have been provided to illustrate themes.

For peer review only

Results

Sample

We recruited 392 women (196 per arm) to the PROSPER clinical trial from 17 breast cancer centres in England. Overall, 67% (n=264/392) of trial participants provided signed consent to be contacted for an interview. In total, we attempted to contact 53 women regarding an interview. Of these, 11 were not contactable, 17 agreed initially for an interview but could not be reached again, and five declined. Ten participants from the intervention arm and ten from the control arm were interviewed from 11 of the 17 study sites (see Table 1).

For peer review only - http://bmjopen.bmj.com/site/about/guidelines.xhtml

Interviews were carried out with 11/44 (25%) physiotherapists (all female) trained to deliver the intervention from six study sites. Ten were interviewed in pairs and one individually. The physiotherapists had treated between one and 16 trial participants (median 5) and were based at hospitals that did not routinely provide postoperative physiotherapy after breast cancer surgery. They were experienced in the management of musculoskeletal conditions but did not work in breast cancer or oncology units. Some physiotherapists had experience of treating breast cancer patients presenting with problems such as restricted shoulder movement preventing the start of radiotherapy.

Table 1 - Study sample (trial participants)

**Characteristic**	**Intervention arm N=10**	**Control arm N=10**
Months since randomisation, mean*(range)*	7 (3-11)	7 (3-12)
Age at randomisation, mean *(range)*	51 (28-69)	60 (44-79)
Age at randomisation		
18-29	1	0
30-39	0	0
40-49	4	2
50-59	3	3
60-69	2	3
70-79	0	2
Ethnicity		
White	9	10
Mixed	1	0
Surgical treatment*		
Mastectomy	4	3
Breast conserving surgery	6	7
Axillary node clearance	10	8
Sentinel lymph node biopsy	3	4
Adjuvant therapy*		
Chemotherapy	9	7
Radiotherapy	9	10

**participants had multiple treatments*

We identified three themes from the data: ‘healing’; ‘being the perfect therapist’; and ‘delivering physiotherapy to breast cancer patients’. Each theme is described below with subthemes. Supporting quotes are provided in Tables 2-4.

For peer review only - http://bmjopen.bmj.com/site/about/guidelines.xhtml

Healing

Reassurance

In the acute period after surgery, all participants felt afraid to move their upper body, and they felt unable to do the exercises prescribed in the Breast Cancer Care information leaflet. Participants allocated to the intervention felt reassured by physiotherapists that they were capable and able to move. They felt reassured that bodily sensations, such as stiffness, were normal and not something to worry about. Physiotherapists felt that they were able to increase participants' confidence in moving their bodies, and that this lifted participants' confidence more broadly. Some described this as giving participants 'permission' to move, which was necessary to prevent movement restrictions in the upper body.

Making progress

This theme refers to physical improvements felt by participants in the intervention arm. This included how far they could stretch and how strong they were. Improvements were measurable and tangible, and participants highlighted the central role of the physiotherapist in creating this sense of progress.

Over time, participants progressed from gentle stretching to more advanced stretching and strengthening exercises as they improved. Progression was fulfilling and rewarding, particularly in the context of cancer treatment where a sense that they were improving or getting better was lacking. To be able to measurably perceive progress in strength and movement helped to restore a sense of bodily autonomy for the women who felt disempowered by cancer treatment. It helped them to feel that they *were* getting better, at least in some way.

Helping myself

During breast cancer treatment, women passively receive treatment [7-9, 30]. One participant described it as being "a professional waiter, you just sit and wait, and you just let everyone do what they're doing" - QR23 (Age 62, Control arm participant).

In collaboration with their physiotherapist, participants receiving the exercise intervention could choose which exercises they performed from a menu, selecting exercises they felt most confident and comfortable doing. The physiotherapists felt that joint-decision making was a patient-centred approach which added to patients' sense of

For peer review only - http://bmjopen.bmj.com/site/about/guidelines.xhtml

ownership and control of their exercises. Physiotherapists and participants both noted that this gave patients an opportunity to be pro-active, taking control of one aspect of their recovery. This sense of responsibility and ownership motivated them to exercise.

This is in contrast to the control arm interviewees. Most control participants spoke about accepting postoperative problems or just waiting for these to improve over time, apart from a few highly motivated individuals who described inventing their own exercises. Wanting to be a 'good' patient and doing as one was told motivated control group participants to follow the exercises on the Breast Cancer Care leaflet. This is in contrast to the sense of self-determination, control, and progress described by participants receiving the intervention.

Looking ahead

Some participants continued to draw on knowledge gained from the intervention to alleviate ongoing problems with tightness and stiffness, and appeared to feel quite confident in managing this in the future. They felt assured that continuing with such activities would help them, and that they would know what to do or where to seek help if required.

Table 2 - Theme 1: Healing

Subtheme	Supporting quotes
Reassurance	*It's quite tender…you don't feel like you ought to be doing it…you feel like it's too soon…I was aching so much that I just thought 'I just can't do this'.* – Qualitative Respondent (QR) 24 (Age 51, Control arm participant)<br><br>*The, er, physiotherapist…was able to tell you whether you were doing things right or wrong or how things were going within your body.* – QR09 (Age 69, Intervention arm participant)<br><br>*They think they're going to split their stitches if they stretch them up. So it's just the reassurance and guidance we give them.* – PT09 (Physiotherapist)<br><br>Interviewer: What do you think they get out of coming to see you?<br>PT02 (Physiotherapist): *I think confidence. Confidence to actually move…confidence to look after themselves, that they can do things*<br><br>*Some people it completely changed their kind of outlook on what they could achieve…it was really encouraging for me to see like you'd given them a new lease of life or like a new hopefulness about what they could achieve in the future.* – PT08 (Physiotherapist)
Making progress	*You could kind of measure it yourself and assess it yourself because you knew how far you could get your arm up…You could feel when, when things started to get a bit better.* – QR08 (Age 43, Intervention arm participant)<br><br>*You saw results and sometimes with your cancer…you don't see results until the end 'til they say 'You're all clear' you are just going through awful, awful, awful praying and hoping…But it is a really positive thing to think 'Oh* something *is getting better'.* – QR12 (Age 55, Intervention arm participant, participant's emphasis)

For peer review only - http://bmjopen.bmj.com/site/about/guidelines.xhtml

For peer review only

	Having those meetings with somebody and seeing the progress that I was making and having her tell me, you know, 'Yeah this is great and now try this' and then having different exercises it kind of made it better for me. – QR13 (Age 28, Intervention arm participant)
Helping myself	*I think it was more than the exercise. I think it was because you were doing something, because so much of um cancer care is being done to you…It was just quite nice to have something proactive for you to do rather than just turn up and have the drugs.* – QR12 (Age 55, Intervention arm participant)<br><br>*That was the biggest thing was that they felt that they were doing something for themselves to try and help their arm with the cancer that we weren't always doing things to them, they had the confidence to do it for themselves.* – PT02 (Physiotherapist)<br><br>*For me, you know, having the same… the same desired outcome as the physiotherapist and* [wife] *you know, kind of, being all, all, all wanting the same thing. And it kind of felt if I did those things then I would eventually achieve it.* – QR08 (Age 43, Intervention arm participant)<br><br>*I think when we were sort of promoting why we think the exercises were useful I talked about self-determination this is something that you can do for yourself and your care…particularly the way it was designed that enabled the patients to say well we could do this exercise or we could do that one.* – PT10 (Physiotherapist)<br><br>Control arm responses in contrast:<br>*It still gets stiff now but you just have to deal with it.* – QR19 (Age 68, Control arm participant)<br><br>*Lifting up now and I can feel the stretching down that left-hand side, but, um, you know I don't know, I suppose it's had trauma.* – QR15 (Age 44, Control arm participant)<br><br>*Well I'd like to think I was a good patient, I started my exercises the day after I came out of hospital.* – QR26 (Age 56, Control arm participant)<br><br>*Just the fact that the hospital gave them you and, you know, they know what they're talking about. You do it because you've been told to.* – QR23 (Age 62, Control arm participant)
Looking ahead	*It's a nice thing to fall back on when I haven't and I think 'Oh this feels a bit tight' then it's like 'Right' get your act into gear and then do it and it does straight away it loosens it.* – QR12 (Age 55, Intervention arm participant)<br><br>*Now I'm just doing the massage for lymphoedema and exercises only if I feel the problem…For example if, if I feel the problem to reach the shelf I'm taking* [the] *band and I might warm it up just do the exercises with the elastic band exactly for this movement.* – QR10 (Age 50, Intervention arm participant)<br><br>*I still go to the gym and there's a really nice instructor there and he's set me a new, um, what do you call it, programme* [for] *strengthening* – QR12 (Age 55, Intervention arm participant)<br><br>*In a couple of months or so, I would like to kind of start using weights so that I can strengthen my arms…It's kind of like building up the strength that I was building towards whilst I was doing the* [PROSPER] *exercises before.* – QR13 (Age 28, Intervention arm participant)

Being the 'perfect' therapist

This theme describes the physiotherapists' reflections on the trial intervention compared to their usual practice and how it enabled them to deliver an optimal service. Specifically, having longer appointment times and an

For peer review only - http://bmjopen.bmj.com/site/about/guidelines.xhtml

emphasis on shared goals and shared decisions, both of which encouraged exercise adherence, were viewed as central. Many of the therapists remarked that they were pleased to be able to offer this service to breast cancer patients because of their previous experience of treating women struggling with chronic immobility, pain, and psychological issues as a result of shoulder problems following breast cancer surgery. Physiotherapists connected this to the broader organisation of the NHS, and the need allocate resources to preventive care.

Table 3 - Theme 2: Being the 'perfect' therapist

Supporting Quotes
It almost like it made you be the perfect physio and the perfect way you should treat patients but you don't always have time to do that. – PT03 (Physiotherapist)
Agreed goals [and] *agreed exercises actually that should be what we're doing anyway that shouldn't be anything radically different but sometimes because of time pressures you don't…If you work more collaboratively with patients there are massive benefits to it and I think it just reinforced that for me.* – PT10 (Physiotherapist)
When you pick up those patients [later] *they come with a lot of emotional baggage and sort of their belief systems and it may have been years since they used their shoulder normally and then you know again if you've got body dysmorphic issues and they've been carrying that around for two years that's a lot more challenging to support.* – PT06 (Physiotherapist)
We get people coming in about two years later and they've never touched their scar, they never saw a physio, they're stiff, their scar's horrible, they've got awful myofascial trigger points and tightness…They still think two years down the line they're going to hurt themselves if they over stretch so if you get them in at the early stage then it's just better…I had a lady who had a mastectomy it was three years later she never went back to work, she never went back to any exercise, she never touched her scar, her mental wellbeing was like absolutely awful when I first started seeing her because she just didn't even know that she could have her life back. – PT01 (Physiotherapist)
I think we work too much reactive in the NHS don't we and I think a direction to move in is work in prevention rather than cure. – PT02 (Physiotherapist)
It makes absolutely no sense you know we're doing all these operations round here where they're just doing small incisions and they routinely see us, but the breast cancer patients who've just had major surgery are just left. It's just madness. – PT01 (Physiotherapist)

For peer review only

Delivering physiotherapy to breast cancer patients

This theme reports views on delivering a new physiotherapist-led intervention for breast cancer patients.

Meeting the needs of breast cancer patients

Participants and physiotherapists suggested that adjuvant treatment, such as chemotherapy, interfered with the patients' ability to maintain the exercise programme. After stopping the exercises when they felt unwell, it was

physically more difficult to start doing the exercises again. Physiotherapists reflected that intervening at this point may have helped encourage and motivate patients to continue.

The physiotherapists noted that patients needed emotional support, and that it was difficult to provide this in a curtained cubicle in an open-plan space, where patients potentially felt more vulnerable. Two therapists felt that physiotherapists should be female as they would better understand the meaning of losing a breast and more able to engage in the emotional and physical work of treating these patients. This issue was not mentioned by the trial participants we interviewed.

Emotional support for physiotherapists

Physiotherapists typically provide emotional support to patients, however, some therapists highlighted particular challenges in relation to this patient group due to the context of cancer treatment, for example, patients were fearful of dying from breast cancer. This was in contrast to their usual caseload which often involved caring for musculoskeletal patients with chronic conditions. The physiotherapists felt they would need emotional support if they worked routinely with breast cancer patients.

Physiotherapists' time, skills, and organisational integration

Delivering the intervention was time-consuming for physiotherapists. Trial appointments were longer than usual and there were doubts about how this could be practically implemented as part of routine NHS clinical care given current time restrictions on appointments. The physiotherapists felt confident in identifying and treating physical shoulder problems, but often expressed a need for training about breast cancer, its treatments, and cancer specific complications. Cording, lymphedema, and seroma were unfamiliar postoperative complications to some physiotherapists until they took part in the trial. Physiotherapists felt disconnected from the surgical or oncology team treating the patient which was challenging. Better integration with the oncology team would have given them greater understanding of the specific patient's treatment schedule as they sometimes felt uncertain about whether the interventions were appropriate at a particular stage of cancer treatment.

Table 4 - Theme 3: Delivering physiotherapy to breast cancer patients

Subtheme	Supporting quotes
Meeting the needs of breast cancer patients	*On day 17 after chemotherapy it has been a struggle…the last three weeks with the first lot of chemo this…*[doing the exercises has] *been a lot harder than I ever anticipated. –* QR11 (Age 49, Intervention arm participant)

For peer review only - http://bmjopen.bmj.com/site/about/guidelines.xhtml

	Maybe three or four times I was not well and I stopped doing the exercises for three or four days if I was ill and after that it was more difficult to do the exercises after. – QR10 (Age 50, Intervention arm participant)<br><br>*A patient would come in for their first appointment and probably just post-surgery and most of them were quite positive had quite a lot of goals… they'd start their chemotherapy and then it was a whole different ball game because it was just kind of managing their fatigue and we struggled to get people back in for appointments.* – PT02 (Physiotherapist)<br><br>*I think one of the things if I was to launch a service based on this intervention I would try and get a private treatment room 'cause we're working in curtained cubicles a lot of the time and I felt that didn't set the tone, I think if you're asking someone to take their bra off then and you can feel y'know curtains move with the best will in the world, not move open necessarily but you have that sense of 'Oh it's just a piece of material between me and God knows who'.* – PT10 (Physiotherapist)<br><br>*They would probably connect better with a female and I was surprised how much women wanted to talk to me about their connection with their breasts so for a lot of them they felt like that was their femininity or that was um a connection to their womanhood and so I think most guys couldn't relate to how that feels so I could get where they were coming from.* – PT08 (Physiotherapist)
Emotional support for physiotherapists	*I am a person who cries quite easily so I was like 'Ok I need to keep things under control myself because I am the professional'… If I was to do it longer term I would need some better kind of guidance and help to deal with that…sometimes I felt a little bit lost.* – PT08 (Physiotherapist)<br><br>*We were lucky because we had each other but there were times where it was upsetting to hear…If we were permanent members of staff in oncology you would be given some…de-briefing or kind of decompression but we were never offered that… both of us have had very close relatives die because of cancer…nobody considered that at all.* – PT06 (Physiotherapist)
Physiotherapists' time, skills, and organisational integration	*I would say giving them the choice of exercise is time consuming which you wouldn't have in real life, you wouldn't have the time.* – PT09 (Physiotherapist)<br><br>*We are MSK* [musculoskeletal] *physios and we know what a tight shoulder is and we know how to get it moving, so actually the assessment and the exercises wasn't so much of a worry, but patients occasionally asked me a question that maybe I couldn't answer…the background behind the cancer, a bit more about the actual surgical techniques they did and why and a little bit more about the reasoning of why lymphedema and cording does actually develop and what it means, I might have benefitted from more training from that aspect.* – PT03 (Physiotherapist)<br><br>*Being able to advise people a bit more around like scar massage or kind of…any of the manual treatments that we could've done and when is right and wrong to use them* [was difficult]. – PT02 (Physiotherapist)<br><br>*There's not necessarily the integration with like the nurses or the lymphedema team, we are quite a separate team from them so I think it does need to be a multi-disciplinary approach and because we're not involved with them it makes it a little bit difficult* [to know] *whether we should or shouldn't be doing those interventions.* – PT03 (Physiotherapist)<br><br>*I sometimes found it difficult to ask about things like chemo, radiotherapy and repeat surgeries because I almost felt like it was something that I should know… I find that, I feel a bit uncomfortable about that, that I think they come in, and expect, and that's what I'd want as a healthcare professional I want them to know what's going on I*

For peer review only - http://bmjopen.bmj.com/site/about/guidelines.xhtml

	shouldn't have to tell you when I am having my chemo or this is happening. – PT05 (Physiotherapist)

Discussion

This qualitative study embedded within a large multicentre clinical trial makes a unique contribution to the literature. Previous studies have explored perceptions of exercise in the context of an exercise intervention [31-33], but this is the first to include the perspectives of both intervention and control group participants, as well as physiotherapists delivering the intervention. We gained multiple perspectives on the same issue, and included all stakeholders in the study. By using qualitative methods, we elicited the particular elements of the intervention which helped motivate participants, and those which were easier or more difficult to deliver in the clinical setting. This intervention is the first early structured physiotherapy-led home-based exercise intervention to be tested in breast cancer patients in the UK. An understanding of the acceptability of the intervention to patients and physiotherapists will inform implementation strategies if the intervention is clinically and cost-effective.

Uncertainty has been identified as a feature of the experience of cancer [34, 26]. Seeing improvement for themselves in terms of strength and stretching stood out in sharp contrast with the uncertainty surrounding cancer and its treatment. Participants also gained a sense of control over their progress, through being involved in choosing exercises, and through taking responsibility for completing their exercises each day. This combination appeared to restore patients' sense of autonomy over their bodies, and improved their wellbeing as they felt less disempowered and hopeless. This echoes previous research which found feelings of increased empowerment when breast cancer patients participated in physical activity during active treatment [35-37]. These experiences contrasted to those in the control group, who did not experience the same sense of empowerment and progress. Specific aspects of the intervention which contributed to this sense of control over and above usual care were the contact with physiotherapists and the reassurance this provided, the sense of progress working through the prescribed programme as exercises increased in difficulty, and the shared decision-making used to select the home exercises. Previous research has found that participating in a group activity can be a way of forgetting about the illness [31]. Our study illustrates this can also be true for home-based or individually supported exercise programmes.

For peer review only - http://bmjopen.bmj.com/site/about/guidelines.xhtml

Being diagnosed with a serious illness such as cancer can cause an individual to lose trust and confidence in their bodily knowledge and of what their bodies are capable of doing [12, 38-40]. The women in this study reported kinesiophobia (fear of movement) in the acute period following surgery, but those in the intervention arm felt the intervention helped them overcome this. Kinesiophobia has been shown to be associated with lymphoedema and greater pain intensity [41, 42]. Physiotherapists were able to reassure patients that their bodily sensations were normal, and gave them confidence to push themselves physically which motivated them to adhere to the programme. The interview data suggested that the role of the physiotherapist in affirming this progress and confidence was crucial. Physiotherapists provided invaluable emotional support, as patients unburdened onto them and shared their fears about the future and their bodies.

The interviewed physiotherapists enjoyed seeing positive improvements in the participants, and felt passionate about delivering what they viewed as high quality care to this patient group. Physiotherapists felt satisfaction in being able to take preventive action against problems arising in the future for these women. Other authors have called for a more proactive model of health care provision for this patient group, and identified the need to improve physiotherapists' confidence in supporting these patients [43]. Our physiotherapists felt that they needed better integration with the rest of the patient's healthcare providers. Other studies have also emphasised the importance of aligning expectations and knowledge about exercise based rehabilitation across the whole cancer care team [5] Challenges to the exercise programme were the side effects of treatment, in particular fatigue, which has been highlighted in other research as a barrier to exercise for breast cancer patients [31]. If a physiotherapist can provide motivation and encouragement during chemotherapy, this may improve adherence to exercise. However, it is also important to ensure that physiotherapists are sympathetic to treatment-related issues, and can tailor programmes during these periods of fatigue [31].

The interviews highlighted considerations for the implementation of an exercise intervention for breast cancer patients. The intervention should be delivered in a private walled room, ideally with a specifically-trained (female) physiotherapist who is part of the multi-disciplinary oncology team caring for the patient. The most important ingredient of the intervention was contact with the physiotherapists, suggesting resources should be focused on training and supporting physiotherapists to provide this care. Some physiotherapists reported feeling upset when treating patients because of the patients' distress or their own experiences of cancer. This suggests that healthcare professionals caring for oncology patients should be given the opportunity of debriefing and emotional support. This is an important consideration when designing future interventions for this patient group.

For peer review only - http://bmjopen.bmj.com/site/about/guidelines.xhtml

In the PROSPER trial, participants underwent a one hour assessment and then subsequent 30-minute follow-up appointments. Routine UK physiotherapy outpatient appointments are often 40 minutes (assessment) and 20 minutes (follow-up). Physiotherapists worked with patients to select the exercises. This may be challenging to deliver in a resource-stretched NHS context. However, longer appointments with physiotherapists, creating shared goals, and making shared decisions about exercises, were viewed as the most important ingredients in the successful delivery of the intervention. Other studies have highlighted how autonomy of choice over exercises may increase motivation and adherence [32, 44]. Additionally, we provided the PROSPER materials in an attractive ring binder with colour photographs, laminated sheets, and provided exercise diary handouts. Patient-participants said the diary was useful as a prompt to remember to do their exercises, and it was helpful to see photographs of the exercises.

Acting more proactively by providing good access to physiotherapy treatment early after, or alongside, breast cancer treatment could help to reduce the number of patients with cancer presenting with musculoskeletal complications [45]. Although our physiotherapist-participants felt very comfortable with aspects of the intervention such as improving shoulder mobility, they expressed a need for greater training, support, and guidance in relation to specific issues such as cording and lymphoedema. The physiotherapists delivering the PROSPER intervention were musculoskeletal specialists with limited experience in treating patients in the acute postoperative period. Physiotherapists in the UK receive little training in rehabilitation following cancer treatment, reflected by the limited centres across the UK with physiotherapists specialised in oncology [45]. Given the increasing number of people surviving cancer and living with the consequences of cancer treatment, there is an urgent need in the UK to upskill physiotherapists in cancer-related rehabilitation to allow patients better access to this type of rehabilitation.

For peer review only

Conclusion

Cancer treatment is an essentially disempowering experience. This study has highlighted how a physiotherapist-led home exercise programme, with built-in progression and shared decision-making, can help patients undergoing breast cancer treatment to feel a restored sense of control over their wellbeing.

For peer review only - http://bmjopen.bmj.com/site/about/guidelines.xhtml

Author statement

JB is Chief Investigator of the PROSPER Trial. JB/SR/EW/HR/BM contributed to the study protocol. SR collected and analysed the data. JB/BM/HR/JB/EW assisted with analysis and interpretation. SR led the writing of the paper, and all other authors contributed to writing and editing.

Funding

Funding for the PROSPER trial was provided by the Health Technology Assessment programme of the National Institute for Health Research (NIHR) (Project Number 13/84/10). JB is supported by NIHR Research Capability Funding via University Hospitals Coventry and Warwickshire. EW is supported by the NIHR Applied Research Collaboration Oxford and Thames Valley.

Conflicts of interest

The authors have no conflicts of interest.

Data availability statement

The datasets generated during and/or analysed during the current study are not publicly available due to the need to protect the identity of participants.

Patient and Public Involvement

Patients partnered with us for the design of the study, the informational material to support the qualitative research, and the burden of the interview from the patient's perspective.

For peer review only

For peer review only - http://bmjopen.bmj.com/site/about/guidelines.xhtml

Acknowledgements

We would like to thank all the physiotherapists and trial participants who took part in this interview study. We dedicate this paper to Elizabeth (Lizzie) Abbey (nee Fort) who passed away on 3rd April 2018. Lizzie helped set up the trial and was a highly valued member of the PROSPER team.

References

1. McNeely ML, Campbell K, Ospina M, Rowe BH, Dabbs K, Klassen TP et al. Exercise interventions for upper-limb dysfunction due to breast cancer treatment. Cochrane Database Syst Rev. 2010(6). doi:10.1002/14651858.CD005211.pub2.
2. Mejdahl MK, Andersen KG, Gärtner R, Kroman N, Kehlet H. Persistent pain and sensory disturbances after treatment for breast cancer: six year nationwide follow-up study. BMJ. 2013;346:f1865.
3. Cardoso F, Kyriakides S, Ohno S, Penault-Llorca F, Poortmans P, Rubio IT et al. Early breast cancer: ESMO Clinical Practice Guidelines for diagnosis, treatment and follow-up. Ann Oncol. 2019. doi:10.1093/annonc/mdz173.
4. Excellence NIfC. Early and locally advanced breast cancer: diagnosis and management NICE guideline [NG101]. 2018. https://www.nice.org.uk/guidance/ng101/chapter/Recommendations. Accessed 31st January 2020.
5. Dennett AM, Harding KE, Reed MS. The challenge of timing: a qualitative study on clinician and patient perspectives about implementing exercise-based rehabilitation in an acute cancer treatment setting. Support Care Cancer. 2020. doi:10.1007/s00520-020-05436-7.
6. Fisher MI, Howell D. The power of empowerment: An ICF-based model to improve self-efficacy and upper extremity function of survivors of breast cancer. Rehabil Oncol. 2010;28(3).
7. Dunn J, Steginga SK. Young women's experience of breast cancer: defining young and identifying concerns. Psychooncology. 2000;9(2):137-46.
8. Thomas-MacLean R. Memories of treatment: the immediacy of breast cancer. Qual Health Res. 2004;14(5):628-43. doi:10.1177/1049732304263658.
9. Little M, Jordens CF, Paul K, Montgomery K, Philipson B. Liminality: a major category of the experience of cancer illness. Soc Sci Med. 1998;47(10):1485-94.
10. McCann L, Illingworth N, Wengström Y, Hubbard G, Kearney N. Transitional experiences of women with breast cancer within the first year following diagnosis. J Clin Nurs. 2010;19(13-14):1969-76.
11. Trusson D, Pilnick A, Roy S. A new normal?: Women's experiences of biographical disruption and liminality following treatment for early stage breast cancer. Soc Sci Med. 2016.
12. Rees S. No one scans you and says 'you're alright now': the experience of embodied risk for young women living with a history of breast cancer. Health, Risk & Society. 2018:1-13. doi:10.1080/13698575.2018.1539468.
13. Bruce J, Williamson E, Lait C, Richmond H, Betteley L, Lall R et al. Randomised controlled trial of exercise to prevent shoulder problems in women undergoing breast cancer treatment: study protocol for the prevention of shoulder problems trial (UK PROSPER). BMJ Open. 2018;8(3):e019078. doi:10.1136/bmjopen-2017-019078.
14. Richmond H, Lait C, Srikesavan C, Williamson E, Moser J, Newman M et al. Development of an exercise intervention for the prevention of musculoskeletal shoulder problems after breast cancer treatment: the prevention of shoulder problems trial (UK PROSPER). BMC Health Serv Res. 2018;18(1):463. doi:10.1186/s12913-018-3280-x.
15. Archer MS. Realist social theory: The morphogenetic approach. Cambridge University Press; 1995.
16. Bhaskar R. A realist theory of science. Routledge; 2013.
17. Braun V, Clarke V. Reflecting on reflexive thematic analysis. Qualitative Research in Sport, Exercise and Health. 2019;11(4):589-97. doi:10.1080/2159676X.2019.1628806.
18. O'Brien BC, Harris IB, Beckman TJ, Reed DA, Cook DAJAM. Standards for reporting qualitative research: a synthesis of recommendations. 2014;89(9):1245-51.
19. Ltd QIP. NVivo qualitative data analysis software. QSR International Pty Ltd. Stokholm, Sweden; 2008.

For peer review only - http://bmjopen.bmj.com/site/about/guidelines.xhtml

20. Guest G, MacQueen KM, Namey EE. Applied thematic analysis. Sage; 2011.
21. Boyatzis RE. Transforming qualitative information: Thematic analysis and code development. sage; 1998.
22. Charmaz K. Constructing grounded theory. Sage; 2014.
23. Braun V, Clarke V. To saturate or not to saturate? Questioning data saturation as a useful concept for thematic analysis and sample-size rationales. Qualitative Research in Sport, Exercise and Health. 2019:1-16. doi:10.1080/2159676X.2019.1704846.
24. Hennink MM, Kaiser BN, Marconi VCJQhr. Code saturation versus meaning saturation: how many interviews are enough? 2017;27(4):591-608.
25. Rees S. A qualitative exploration of the meaning of the term "survivor" to young women living with a history of breast cancer. European journal of cancer care. 2018;27(3):e12847.
26. Rees S. 'Am I really gonna go sixty years without getting cancer again?'Uncertainty and liminality in young women's accounts of living with a history of breast cancer. Health:. 2017;21(3):241–58. doi:10.1177/1363459316677628.
27. Rees S, Young A. The experiences and perceptions of women diagnosed with breast cancer during pregnancy. Asia-Pacific journal of oncology nursing. 2016;3(3):252-8. doi:10.4103/2347-5625.189814.
28. Hutchinson A, Rees S, Young A, Maraveyas A, Date K, Johnson MJ. Oral anticoagulation is preferable to injected, but only if it is safe and effective: An interview study of patient and carer experience of oral and injected anticoagulant therapy for cancer-associated thrombosis in the select-d trial. Palliat Med. 2019;33(5):510-7.
29. Lincoln YS, Guba EG. But is it rigorous? Trustworthiness and authenticity in naturalistic evaluation. New directions for program evaluation. 1986;1986(30):73-84.
30. Trusson D. Living with a new normal: women's experiences following treatment for early-stage breast cancer or DCIS: University of Nottingham; 2013.
31. Browall M, Mijwel S, Rundqvist H, Wengstrom Y. Physical Activity During and After Adjuvant Treatment for Breast Cancer: An Integrative Review of Women's Experiences. Integr Cancer Ther. 2018;17(1):16-30. doi:10.1177/1534735416683807.
32. Ingram C, Wessel J, Courneya KS. Women's perceptions of home-based exercise performed during adjuvant chemotherapy for breast cancer. Eur J Oncol Nurs. 2010;14(3):238-43. doi:10.1016/j.ejon.2010.01.027.
33. Luoma ML, Hakamies-Blomqvist L, Blomqvist C, Nikander R, Gustavsson-Lilius M, Saarto T. Experiences of breast cancer survivors participating in a tailored exercise intervention -a qualitative study. Anticancer Res. 2014;34(3):1193-9.
34. Halliday LE, Boughton MA, Kerridge I. Mothering and self-othering: The impact of uncertain reproductive capability in young women after hematological malignancy. Health Care Women Int. 2014;35(3):249-65.
35. Husebo AM, Karlsen B, Allan H, Soreide JA, Bru E. Factors perceived to influence exercise adherence in women with breast cancer participating in an exercise programme during adjuvant chemotherapy: a focus group study. J Clin Nurs. 2015;24(3-4):500-10. doi:10.1111/jocn.12633.
36. Crane-Okada R, Kiger H, Anderson NL, Carroll-Johnson RM, Sugerman F, Shapiro SL et al. Participant perceptions of a mindful movement program for older women with breast cancer: focus group results. Cancer Nurs. 2012;35(3):E1-10. doi:10.1097/NCC.0b013e31822539c5.
37. Bulmer SM, Howell J, Ackerman L, Fedric R. Women's perceived benefits of exercise during and after breast cancer treatment. Women Health. 2012;52(8):771-87. doi:10.1080/03630242.2012.725707.
38. Williams SJ. The vicissitudes of embodiment across the chronic illness trajectory. Body & Society. 1996;2(2):23-47.
39. Lindwall L, Bergbom I. The altered body after breast cancer surgery. International Journal of Qualitative Studies on Health and Well-being. 2009;4(4):280-7.
40. Crouch M, McKenzie H. Social realities of loss and suffering following mastectomy. Health:. 2000;4(2):196-215.
41. Gencay Can A, Can SS, Ekşioğlu E, Çakcı FA. Is kinesiophobia associated with lymphedema, upper extremity function, and psychological morbidity in breast cancer survivors? Turkish journal of physical medicine and rehabilitation. 2018;65(2):139-46. doi:10.5606/tftrd.2019.2585.
42. Martinez-Calderon J, Struyf F, Meeus M, Luque-Suarez A. The association between pain beliefs and pain intensity and/or disability in people with shoulder pain: A systematic review. Musculoskelet Sci Pract. 2018;37:29-57. doi:10.1016/j.msksp.2018.06.010.
43. Levangie PK, Santasier AM, Stout NL, Pfalzer L. A qualitative assessment of upper quarter dysfunction reported by physical therapists treated for breast cancer or treating breast cancer sequelae. Support Care Cancer. 2011;19(9):1367-78. doi:10.1007/s00520-010-0959-x.
44. Jones LW, Courneya KS, Fairey AS, Mackey JR. Does the theory of planned behavior mediate the effects of an oncologist's recommendation to exercise in newly diagnosed breast cancer survivors? Results from a randomized controlled trial. Health Psychol. 2005;24(2):189.

For peer review only - http://bmjopen.bmj.com/site/about/guidelines.xhtml

45. Robinson M, Ward L, Mehanna H, Paleri V, Winter SC. Provision of physiotherapy rehabilitation following neck dissection in the UK. The Journal of Laryngology & Otology. 2018;132(7):624-7. doi:10.1017/S0022215118000671.

For peer review only

For peer review only - http://bmjopen.bmj.com/site/about/guidelines.xhtml

Reporting checklist for qualitative study.

Based on the SRQR guidelines.

Instructions to authors

Complete this checklist by entering the page numbers from your manuscript where readers will find each of the items listed below.

Your article may not currently address all the items on the checklist. Please modify your text to include the missing information. If you are certain that an item does not apply, please write "n/a" and provide a short explanation.

Upload your completed checklist as an extra file when you submit to a journal.

In your methods section, say that you used the SRQRreporting guidelines, and cite them as:

O'Brien BC, Harris IB, Beckman TJ, Reed DA, Cook DA. Standards for reporting qualitative research: a synthesis of recommendations. Acad Med. 2014;89(9):1245-1251.

		Reporting Item	Page Number
**Title**			
	#1	Concise description of the nature and topic of the study identifying the study as qualitative or indicating the approach (e.g. ethnography, grounded theory) or data collection methods (e.g. interview, focus group) is recommended	
**Abstract**			
	#2	Summary of the key elements of the study using the abstract format of the intended publication; typically includes background, purpose, methods, results and conclusions	
**Introduction**			
Problem formulation	#3	Description and signifcance of the problem / phenomenon studied: review of relevant theory and empirical work; problem statement	
Purpose or research question	#4	Purpose of the study and specific objectives or questions	

For peer review only

Methods

Qualitative approach and research paradigm	#5	Qualitative approach (e.g. ethnography, grounded theory, case study, phenomenolgy, narrative research) and guiding theory if appropriate; identifying the research paradigm (e.g. postpositivist, constructivist / interpretivist) is also recommended; rationale. The rationale should briefly discuss the justification for choosing that theory, approach, method or technique rather than other options available; the assumptions and limitations implicit in those choices and how those choices influence study conclusions and transferability. As appropriate the rationale for several items might be discussed together.
Researcher characteristics and reflexivity	#6	Researchers' characteristics that may influence the research, including personal attributes, qualifications / experience, relationship with participants, assumptions and / or presuppositions; potential or actual interaction between researchers' characteristics and the research questions, approach, methods, results and / or transferability
Context	#7	Setting / site and salient contextual factors; rationale
Sampling strategy	#8	How and why research participants, documents, or events were selected; criteria for deciding when no further sampling was necessary (e.g. sampling saturation); rationale
Ethical issues pertaining to human subjects	#9	Documentation of approval by an appropriate ethics review board and participant consent, or explanation for lack thereof; other confidentiality and data security issues
Data collection methods	#10	Types of data collected; details of data collection procedures including (as appropriate) start and stop dates of data collection and analysis, iterative process, triangulation of sources / methods, and modification of procedures in response to evolving study findings; rationale
Data collection instruments and technologies	#11	Description of instruments (e.g. interview guides, questionnaires) and devices (e.g. audio recorders) used for data collection; if / how the instruments(s) changed over the course of the study
Units of study	#12	Number and relevant characteristics of participants, documents, or events included in the study; level of participation (could be

For peer review only

		reported in results)
Data processing	#13	Methods for processing data prior to and during analysis, including transcription, data entry, data management and security, verification of data integrity, data coding, and anonymisation / deidentification of excerpts
Data analysis	#14	Process by which inferences, themes, etc. were identified and developed, including the researchers involved in data analysis; usually references a specific paradigm or approach; rationale
Techniques to enhance trustworthiness	#15	Techniques to enhance trustworthiness and credibility of data analysis (e.g. member checking, audit trail, triangulation); rationale
**Results/findings**		
Syntheses and interpretation	#16	Main findings (e.g. interpretations, inferences, and themes); might include development of a theory or model, or integration with prior research or theory
Links to empirical data	#17	Evidence (e.g. quotes, field notes, text excerpts, photographs) to substantiate analytic findings
**Discussion**		
Intergration with prior work, implications, transferability and contribution(s) to the field	#18	Short summary of main findings; explanation of how findings and conclusions connect to, support, elaborate on, or challenge conclusions of earlier scholarship; discussion of scope of application / generalizability; identification of unique contributions(s) to scholarship in a discipline or field
Limitations	#19	Trustworthiness and limitations of findings
**Other**		
Conflicts of interest	#20	Potential sources of influence of perceived influence on study conduct and conclusions; how these were managed
Funding	#21	Sources of funding and other support; role of funders in data collection, interpretation and reporting

None The SRQR checklist is distributed with permission of Wolters Kluwer © 2014 by the Association of American Medical Colleges. This checklist can be completed online using https://www.goodreports.org/, a tool made by the EQUATOR Network in collaboration with Penelope.ai

For peer review only

BMJ Open

The role of physiotherapy in supporting recovery from breast cancer treatment: A qualitative study embedded within the UK PROSPER trial

Journal:	*BMJ Open*
Manuscript ID	bmjopen-2020-040116.R1
Article Type:	Original research
Date Submitted by the Author:	06-Oct-2020
Complete List of Authors:	Rees, Sophie; University of Warwick, Warwick Clinical Trials Unit<br>Mazuquin, Bruno; University of Warwick, Warwick Clinical Trials Unit; Manchester Metropolitan University, Faculty of Health, Psychology and Social Care<br>Richmond, Helen ; University of Warwick, Warwick Clinical Trials Unit; Memorial University of Newfoundland, Faculty of Medicine<br>Williamson, Esther; Nuffield Department of Orthopaedics, Rheumatology and Musculoskeletal Sciences, University of Oxford, United Kingdom. , Centre for Rehabilitation Research in Oxford<br>Bruce, Julie; University of Warwick, Warwick Clinical Trials Unit<br>-, UK PROSPER Study Group; University of Warwick, Warwick Clinical Trials Unit
**Primary Subject Heading**:	Oncology
Secondary Subject Heading:	Qualitative research, Surgery
Keywords:	QUALITATIVE RESEARCH, Breast surgery < SURGERY, PREVENTIVE MEDICINE

SCHOLARONE™
Manuscripts

I, the Submitting Author has the right to grant and does grant on behalf of all authors of the Work (as defined in the below author licence), an exclusive licence and/or a non-exclusive licence for contributions from authors who are: i) UK Crown employees; ii) where BMJ has agreed a CC-BY licence shall apply, and/or iii) in accordance with the terms applicable for US Federal Government officers or employees acting as part of their official duties; on a worldwide, perpetual, irrevocable, royalty-free basis to BMJ Publishing Group Ltd ("BMJ") its licensees and where the relevant Journal is co-owned by BMJ to the co-owners of the Journal, to publish the Work in this journal and any other BMJ products and to exploit all rights, as set out in our licence.

The Submitting Author accepts and understands that any supply made under these terms is made by BMJ to the Submitting Author unless you are acting as an employee on behalf of your employer or a postgraduate student of an affiliated institution which is paying any applicable article publishing charge ("APC") for Open Access articles. Where the Submitting Author wishes to make the Work available on an Open Access basis (and intends to pay the relevant APC), the terms of reuse of such Open Access shall be governed by a Creative Commons licence – details of these licences and which Creative Commons licence will apply to this Work are set out in our licence referred to above.

Other than as permitted in any relevant BMJ Author's Self Archiving Policies, I confirm this Work has not been accepted for publication elsewhere, is not being considered for publication elsewhere and does not duplicate material already published. I confirm all authors consent to publication of this Work and authorise the granting of this licence.

For peer review only

The role of physiotherapy in supporting recovery from breast cancer treatment: A qualitative study embedded within the UK PROSPER trial

Rees S,[1] Mazuquin B,[1,2] Richmond H,[1,3] Williamson E,[4] Bruce J.[1] on behalf of the UK PROSPER Study Group[1]

[1] *Warwick Clinical Trials Unit, University of Warwick Medical School, Coventry, UK CV4 7AL*

[2] *Faculty of Health, Psychology and Social Care, Manchester Metropolitan University*

[3] *Faculty of Medicine, Memorial University of Newfoundland, St John's, Canada*

[4] *The Centre for Rehabilitation Research, Nuffield Department of Orthopaedics, Rheumatology and Musculoskeletal Sciences, University of Oxford*

Corresponding author:
Dr Sophie Rees
S.Rees.1@warwick.ac.uk
02476 151 135

For peer review only

Abstract

Objectives: To explore breast cancer patients' experience of taking part in an early physiotherapy-led exercise intervention compared with the experiences of those receiving usual care. To understand physiotherapists' experience of delivering the trial intervention. To explore issues related to the implementation of the PROSPER programme from participant and physiotherapist perspective.

Design: Qualitative audio-recorded semi-structured interviews with thematic analysis.

Setting: UK National Health Service (NHS)

Participants: Twenty participants at high risk of shoulder problems after breast cancer surgery, recruited to the UK Prevention of Shoulder Problems (PROSPER) Trial (ten each from the intervention arm and control arm respectively); and eleven physiotherapists who delivered the intervention. Trial participants were sampled using convenience sampling. Physiotherapists were purposively sampled from high and low recruiting sites.

Results: Participants described that the PROSPER exercise intervention helped them feel confident in what their body could do, and helped them regain a sense of control in the context of cancer treatment which was largely disempowering. Control arm participants expressed less of a sense of control over their wellbeing.

Physiotherapists found the exercise intervention enjoyable to deliver and felt it was valuable to their patients. The extra time allocated to patients during intervention delivery made physiotherapists feel they were providing optimal care, being the ‘perfect physio’. Lessons were learned about the implementation of a complex exercise intervention for breast cancer patients and the issues raised will inform the development of a future implementation strategy.

Conclusions: A physiotherapist-delivered early supported exercise intervention with integrated behavioural strategies helped women at risk of shoulder problems following breast cancer treatment to regain control and feel more confident in their ability to mobilise their arm post-surgery. A physiotherapist-delivered early supported exercise intervention with integrated behavioural strategies may address the sense of powerlessness that many patients experience during cancer treatment.

Keywords: *breast cancer; physiotherapy; rehabilitation; prevention; shoulder; qualitative research*

Acknowledgements

This study was embedded within a multicentre UK randomised controlled trial (ISCRTN35358984) and was funded by the National Institute for Health Research Health Technology Assessment Programme (HTA 13/84/10). The research team would like to thank the trial participants and physiotherapists who participated in the qualitative study.

Word count: 6,259 (due to quotes being included in word count)

For peer review only

Article Summary

Strengths and limitations of this study

- Interviewing multiple groups (intervention arm, control arm participants, and physiotherapists) in this study enabled us to triangulate the data and explore experiences from multiple perspectives.
- We note that the participants we interviewed were a particularly motivated group, and it is possible we did not capture some of the challenges which other, less motivated, women may have experienced.
- We obtained consent to be approached for interview prior to randomisation, independent of treatment allocation, in an attempt to minimise bias. We tried to minimise the risk of social desirability bias by asking neutral questions and explaining there were no right or wrong answers
- We used a convenience sampling approach, which is a potential weakness of this study as it may have resulted in a lack of diversity amongst participants.
- Our sample was overwhelmingly white, with only one of the participants identifying as another ethnic identity. Findings may not reflect the experiences of black, Asian and other minority ethnic groups.

Introduction

Treatment to the chest and axilla for breast cancer can result in upper body problems, such as reduced range of movement in the shoulder, muscle weakness, pain, lymphoedema, and functional limitations [1, 2]. These problems can impact on ability to carry out activities of daily living, and may persist for many years after treatment [1, 2]. Exercise in the acute phase following breast cancer surgery may improve shoulder function in women at high risk of shoulder problems [1]. Guidelines state that breast cancer patients should be referred to physiotherapy when indicated [3, 4], however, in the UK NHS this is not routine practice. There is a need for a proactive model of care which encourages early exercise-based rehabilitation and provides physiotherapists with resources to inform their practice [5]. Loss of a sense of control, loss of self-identity, and alienation from their bodies during and after treatment are often reported by breast cancer patients [6-12]. It has been proposed that improving women's self-efficacy through physical rehabilitation may improve their quality of life [6]. Lack of knowledge about exercise, and the experience of cancer-related fatigue were identified as obstacles to exercise in a recent

For peer review only - http://bmjopen.bmj.com/site/about/guidelines.xhtml

For peer review only

study of Korean patients [13]. A recent systematic review of the qualitative literature identified six studies of mixed quality reporting the experiences of women living beyond breast cancer of participating in a supervised exercise intervention [14]. These studies all reported on group interventions, and the findings suggest that the group element may be beneficial. Little is known about the experiences of this patient group participating in individual supported exercise intervention. There has also been little published regarding the experiences of professionals delivering exercise interventions to this patient group. One recent study reported a lack of confidence amongst physiotherapists in treating cancer patients, but respondents felt confidence grew with practice [15]. Little is known about how physiotherapists feel about the feasibility of implementing a service for people with breast cancer. This is important so that we can address challenges and issues when designing services.

The UK Prevention Of Shoulder Problems (PROSPER) Trial evaluated the clinical and cost-effectiveness of an early supported home-based physiotherapist-led exercise intervention in women with newly diagnosed breast cancer at higher risk of developing shoulder problems after treatment [16, 17]. A description of the intervention and trial protocol have been published [16, 17]. This paper reports the findings of the UK PROSPER trial embedded qualitative study.

The aims of the qualitative study were:

- To understand the acceptability of the exercise intervention to participants
- To explore how the exercise intervention or control affected their experiences of recovery after cancer treatment.
- To investigate the experiences of physiotherapists delivering the exercise intervention.
- To explore participants' and physiotherapists' perspectives on issues related to the implementation of the PROSPER programme to inform future plans for implementation. .

Figure 1 illustrates the pathway of participants through the trial and embedded qualitative study.

Figure 1 - Participant pathway through trial and qualitative study

For peer review only - http://bmjopen.bmj.com/site/about/guidelines.xhtml

Methods

Methodology

The study was underpinned by critical realism, assuming that an underlying reality is experienced and given meaning by individuals [18, 19]. To meet the study aims, we conducted qualitative semi-structured interviews with reflexive thematic analysis [20]. This allowed for exploration, depth, and understanding of the experiences of trial participants, thus taking an interpretive 'sense-making' approach rather than hypothesis-testing or confirmatory approach. We used the SRQR reporting guidelines checklist [21].

Sampling and recruitment

Trial participant interviews

On recruitment to the trial, we offered all trial participants the option to take part in an interview at a later date (see Figure 1). We recorded signed consent to be approached for interview and this formed our sampling frame for the qualitative study. Women in the intervention arm were approached after they were discharged from physiotherapy to avoid contamination bias. The researcher (SR) telephoned participants to invite them to interview, and, if they expressed an interest, participants were sent an information sheet and interview consent form.

After conducting and analysing seven interviews with intervention participants, we decided to interview control arm participants, to compare their experiences. We used our database of those who had consented pre-randomisation to select a sample comparable to the intervention sample in terms of time since randomisation, so that women were at similar stages of postoperative treatment and could reflect back over their experiences of recovery.

Physiotherapist interviews

All physiotherapists delivering the intervention were informed about the interview study. We then sampled physiotherapists from low and high recruiting trial sites to allow exploration of different perspectives on intervention delivery. Therapists were approached by the researcher via email or telephone.

For peer review only - http://bmjopen.bmj.com/site/about/guidelines.xhtml

For peer review only

Data collection

Flexible topic guides were developed by the research team and with breast cancer patients, based on the aims of the study and relevant literature. One-off interviews were conducted either at the participant's home, by telephone (trial participants), or in a private room at their place of work (physiotherapists). Physiotherapists who worked together were interviewed in pairs. The physiotherapists who volunteered for the interviews worked closely together. Interviewing them in pairs allowed physiotherapists to share and reflect on their experiences, and aided recall where they had only treated a small number of participants, for example. It is possible that interviewing them in pairs could have affected their responses, but participants were remarkably candid about the challenges they experienced, thus we were not concerned that this was happening. Only the researcher and interviewees were present. All interviews were audio-recorded. We took study materials (physiotherapy manual, participant materials) into the interview to aid recall and discussion. Informed consent was gained before the interview began. The study was approved by the National Research Ethics Service Committee West Midlands Solihull on 20th July 2015 (Ref no. 15/WM/0224),

Data analysis

Interviews were transcribed verbatim, checked for accuracy and anonymity by the researcher (SR), and then uploaded to QSR NVivo Pro 11 [22]. Thematic analysis [23, 24] was conducted by SR and managed in NVivo. Interview transcripts were 'coded', where sections of text are assigned a descriptive label, producing dozens of codes per interview. These codes were then grouped into categories, and these were then grouped further into themes. Analysis began alongside data collection. The research team met regularly to discuss emerging findings and the evolving analysis [25]. Saturation in this study meant that we had enough data to understand each of the identified categories and themes, rather than that there was 'nothing new' to be found [26, 27]. We reached saturation after fifteen trial participant interviews, and five physiotherapist interviews.

Reflexivity and rigour

Interviews were conducted sensitively by a female researcher experienced in interviewing cancer patients (SR) [12, 28-31]. The evolving analysis was discussed with the research team (SR/JB/HR/BM). SR is a social scientist with expertise in qualitative research with people with health conditions, including breast cancer, and healthcare professionals. HR and BM are researchers and physiotherapists. JB is a trialist and PROSPER Chief Investigator,

For peer review only - http://bmjopen.bmj.com/site/about/guidelines.xhtml

she did not influence the qualitative study findings, but provided important contextual details regarding the trial and intervention. We were careful to conduct balanced interviews, without assuming that the trial participants and physiotherapists would have positive views of the intervention. SR reminded interviewees throughout that she was not involved in the development of the intervention, and welcomed their honest views. Rigour was assessed using Lincoln and Guba's conceptualisation of trustworthiness [32]. SR collected the data and was immersed in the data during analysis. Quotes have been provided to illustrate themes.

Results

Sample

We recruited 392 women (196 per arm) to the PROSPER clinical trial from 17 breast cancer centres in England. Overall, 67% (n=264/392) of trial participants provided signed consent to be contacted for an interview. In total, we attempted to contact 53 women regarding an interview. Of these, 11 were not contactable, 17 agreed initially for an interview but could not be reached again, and five declined. Ten participants from the intervention arm and ten from the control arm were interviewed from 11 of the 17 study sites (see Table 1). There were no apparent differences between the sites regarding the issues raised by trial participants and therapists. We had a good range in terms of size and rural/urban sites across the 11 sites represented in the interview study.

Interviews were carried out with 11/44 (25%) physiotherapists (all female) from six study sites. All therapists had attended a training day which included prescribing the exercises as well as behaviour change techniques to encourage adherence with the programme. Motivational Interviewing techniques were included along with case studies to demonstrate putting these skills into practice. Ten were interviewed in pairs and one individually. The physiotherapists had treated between one and 16 trial participants (median 5) and were based at hospitals that did not routinely provide postoperative physiotherapy after breast cancer surgery. They were experienced in the management of musculoskeletal conditions but did not currently work in breast cancer or oncology units. Some physiotherapists had experience of treating breast cancer patients presenting with problems such as restricted shoulder movement preventing the start of radiotherapy. One physiotherapist had past experience working on a cancer inpatient ward.

For peer review only - http://bmjopen.bmj.com/site/about/guidelines.xhtml

Table 1 - Study sample (trial participants)

Characteristic	Intervention arm N=10	Control arm N=10
Months since randomisation, mean *(range)*	7 (3-11)	7 (3-12)
Age at randomisation, mean *(range)*	51 (28-69)	60 (44-79)
Age at randomisation		
18-29	1	0
30-39	0	0
40-49	4	2
50-59	3	3
60-69	2	3
70-79	0	2
Ethnicity		
White	9	10
Mixed	1	0
Surgical treatment*		
Mastectomy	4	3
Breast conserving surgery	6	7
Axillary node clearance	10	8
Sentinel lymph node biopsy	3	4
Adjuvant therapy*		
Chemotherapy	9	7
Radiotherapy	9	10

**participants had multiple treatments*

We identified three themes from the data: 'healing'; 'being the perfect therapist'; and 'delivering physiotherapy to breast cancer patients'. The themes and subthemes are illustrated by participant group in Table 2, and each theme is described below with subthemes.

For peer review only

Table 2 - Illustration of themes and subthemes by participant group

Theme	Subtheme	Intervention group	Control group	Physiotherapists
Healing	Reassurance	*The physiotherapist was able to tell you whether you were doing things right or wrong*	*It's quite tender…you don't feel like you ought to be doing it…you feel like it's too soon*	*They think they're going to split their stitches…it's just the reassurance we give*
	Making progress	*You saw results and sometimes with your cancer…you don't see results until the end*	*I'm tender but I suppose that will go*	
	Helping myself	*I think it was because you were doing something, because so much of cancer care is being done to you*	*It still gets stiff now but you just have to deal with it.*	*This is something that you can do for yourself and your care*
	Looking ahead	*Now I'm just doing the massage for lymphoedema and exercises only if I feel the problem*		
Being the 'perfect' therapist		*she asked me how I felt and it was very much about me and my progress*		*It almost like it made you be the perfect physio and the perfect way you should treat patients but you don't always have time to do that*
Delivering physiotherapy to breast cancer patients	Meeting the needs of breast cancer patients	*Maybe three or four times I was not well and I stopped doing the exercises for three or four days if I was ill and after that it was more difficult to do the exercises after*		*they'd start their chemotherapy and then it was a whole different ball game because it was just kind of managing their fatigue and we struggled to get people back in for appointments*
	Emotional support for physiotherapists			*there were times where it was upsetting to hear*
	Physiotherapists' time, skills, and organisational integration			*I would say giving them the choice of exercise is time consuming which you wouldn't have in real life, you wouldn't have the time*<br><br>*There's not necessarily the integration with like the nurses or the lymphedema team, we are quite a separate team*<br><br>*Being able to advise people a bit more around like scar massage or kind of…any of the manual treatments that we could've done and when is right and wrong to use them* [was difficult]

For peer review only - http://bmjopen.bmj.com/site/about/guidelines.xhtml

Healing

Reassurance

In the acute period after surgery, participants from both the intervention and control groups reported feeling afraid to move their upper body. This is known as kinesiophobia [33, 34]. They felt unable to do the exercises prescribed in the Breast Cancer Care information leaflet.

> *It's quite tender…you don't feel like you ought to be doing it…you feel like it's too soon…I was aching so much that I just thought 'I just can't do this'.* – Qualitative Respondent (QR24) (Age 51, Control arm participant)

> *Because you don't know whether it's good or not, do you know what I mean, you don't know if you're doing well or not or if this is where you would be, you know, or you should be and it was quite nice because you got 'oh no you're doing really well' or 'oh yeah that will be tight' and it was that… it was quite nice to have the feedback* – QR12 (Age 55, Intervention arm participant)

> *They think they're going to split their stitches if they stretch them up. So it's just the reassurance and guidance we give them.* – PT09 (Physiotherapist)

Participants allocated to the intervention arm subsequently felt reassured by physiotherapists that they were capable and able to move. They felt reassured that bodily sensations, such as stiffness, were normal and not something to worry about. Physiotherapists felt that they were able to increase participants' confidence in moving their bodies, and that this lifted participants' confidence more broadly.

> *The, er, physiotherapist…was able to tell you whether you were doing things right or wrong or how things were going within your body.* – QR09 (Age 69, Intervention arm participant)

> Interviewer: What do you think they get out of coming to see you?
>
> PT02 (Physiotherapist): *I think confidence. Confidence to actually move…confidence to look after themselves, that they can do things*

For peer review only - http://bmjopen.bmj.com/site/about/guidelines.xhtml

> *Some people it completely changed their kind of outlook on what they could achieve…it was really encouraging for me to see like you'd given them a new lease of life or like a new hopefulness about what they could achieve in the future.* – PT08 (Physiotherapist)

Some described this as giving participants 'permission' to move, which was necessary to prevent movement restrictions in the upper body.

Making progress

This theme refers to physical improvements felt by participants in the intervention arm. This included how far they could stretch and how strong they were. Improvements were measurable and tangible, and participants highlighted the central role of the physiotherapist in creating this sense of progress.

> *You saw results and sometimes with your cancer…you don't see results until the end 'til they say 'You're all clear' you are just going through awful, awful, awful praying and hoping…But it is a really positive thing to think 'Oh* something *is getting better'.* – QR12 (Age 55, Intervention arm participant, participant's emphasis)

> *You could kind of measure it yourself and assess it yourself because you knew how far you could get your arm up…You could feel when, when things started to get a bit better.* – QR08 (Age 43, Intervention arm participant)

> *Having those meetings with somebody and seeing the progress that I was making and having her tell me, you know, 'Yeah this is great and now try this' and then having different exercises it kind of made it better for me.* – QR13 (Age 28, Intervention arm participant)

Where intervention participants spoke about the improvement they felt in the months following their surgery, control arm participants also spoke about improvement, but for them this remained an ongoing process even more than 12 months on.

> *when I'm washing myself and, and if I touch myself I'm tender but I suppose that will go* – QR17 (Age 67, Control arm participant)

> *I have still got a seroma on my chest which is a bit of a nuisance which is, um, sort of swelling of fluid isn't it. Um, it's less than it was and I think it's gradually going 'cause it was enormous at the very beginning but it's getting less* – QR16 (age 79, Control arm participant)

For peer review only - http://bmjopen.bmj.com/site/about/guidelines.xhtml

Over time, intervention participants progressed from gentle stretching to more advanced stretching and strengthening exercises as they improved. Graduating to harder exercises gave them a sense of achievement.

> *When we would do the exercises and when we would move the kind of categories in the folder that was given, that made me feel good and made me want to kind of continue.* – QR13 (Age 28, Intervention arm participant)

Progression was fulfilling and rewarding, particularly in the context of cancer treatment where a sense that they were improving or getting better was lacking. To be able to measurably perceive progress in strength and movement helped to restore a sense of bodily autonomy for the women who felt disempowered by cancer treatment. During this profoundly difficult time of undergoing cancer treatment, feeling improvement and graduating to harder exercises helped them to feel that they *were* getting better, at least in some way.

Helping myself

During breast cancer treatment, women passively receive treatment [7-9, 35]. One participant described it as being "a professional waiter, you just sit and wait, and you just let everyone do what they're doing" - QR23 **(**Age 62, Control arm participant).

In collaboration with their physiotherapist, participants receiving the exercise intervention could choose which exercises they performed from a menu, selecting exercises they felt most confident and comfortable doing. The physiotherapists felt that joint-decision making was a patient-centred approach which added to patients' sense of ownership and control of their exercises. Physiotherapists and participants both noted that this gave patients an opportunity to be pro-active, taking control of one aspect of their recovery. This sense of responsibility and ownership motivated them to exercise.

> *I think it was more than the exercise. I think it was because you were doing something, because so much of um cancer care is being done to you…It was just quite nice to have something proactive for you to do rather than just turn up and have the drugs.* – QR12 (Age 55, Intervention arm participant*)*

> *That was the biggest thing was that they felt that they were doing something for themselves to try and help their arm with the cancer that we weren't always doing things to them, they had the confidence to do it for themselves.* – PT02 (Physiotherapist)

For peer review only - http://bmjopen.bmj.com/site/about/guidelines.xhtml

I think when we were sort of promoting why we think the exercises were useful I talked about self-determination this is something that you can do for yourself and your care…particularly the way it was designed that enabled the patients to say well we could do this exercise or we could do that one. – PT10 (Physiotherapist)

This was a key difference to the control arm interviewees. Most control arm participants spoke about accepting postoperative problems or just waiting for them to improve over time, apart from a few highly motivated individuals who described inventing their own exercises.

It still gets stiff now but you just have to deal with it. – QR19 (Age 68, Control arm participant)

Lifting up now and I can feel the stretching down that left-hand side, but, um, you know I don't know, I suppose it's had trauma. – QR15 (Age 44, Control arm participant)

The tightness on my chest does limit movement sometimes and it's sort of more of a discomfort than a pain and just a blessed nuisance really but everyone I've seen says it's normal that they take a while and it's nothing they can do so it will just go when it's ready I suppose and I kind of live with it – QR17 (Age 67, Control arm participant)

Wanting to be a 'good' patient and doing as one was told motivated control group participants to follow the exercises on the Breast Cancer Care leaflet.

Well I'd like to think I was a good patient, I started my exercises the day after I came out of hospital. – QR26 (Age 56, Control arm participant)

Just the fact that the hospital gave them you and, you know, they know what they're talking about. You do it because you've been told to. – QR23 (Age 62, Control arm participant)

This is in contrast to the sense of self-determination, control, and progress described by participants receiving the intervention.

Looking ahead

Some participants continued to draw on knowledge gained from the intervention to alleviate ongoing problems with tightness and stiffness, and appeared to feel quite confident in managing this in the future.

For peer review only - http://bmjopen.bmj.com/site/about/guidelines.xhtml

It's a nice thing to fall back on when I haven't and I think 'Oh this feels a bit tight' then it's like 'Right' get your act into gear and then do it and it does straight away it loosens it. – QR12 (Age 55, Intervention arm participant)

Now I'm just doing the massage for lymphoedema and exercises only if I feel the problem…For example if, if I feel the problem to reach the shelf I'm taking [the] *band and I might warm it up just do the exercises with the elastic band exactly for this movement.* – QR10 (Age 50, Intervention arm participant)

I still go to the gym and there's a really nice instructor there and he's set me a new, um, what do you call it, programme [for] *strengthening* – QR12 (Age 55, Intervention arm participant)

In a couple of months or so, I would like to kind of start using weights so that I can strengthen my arms…It's kind of like building up the strength that I was building towards whilst I was doing the [PROSPER] *exercises before.* – QR13 (Age 28, Intervention arm participant)

They felt assured that continuing with such activities would help them, and that they would know what to do or where to seek help if required.

Being the 'perfect' therapist

This theme describes the physiotherapists' perspectives on the trial intervention compared to their usual practice and how it enabled them to deliver an optimal service.

It almost like it made you be the perfect physio and the perfect way you should treat patients but you don't always have time to do that. – PT03 (Physiotherapist)

Agreed goals [and] *agreed exercises actually that should be what we're doing anyway that shouldn't be anything radically different but sometimes because of time pressures you don't…If you work more collaboratively with patients there are massive benefits to it and I think it just reinforced that for me.* – PT10 (Physiotherapist)

For peer review only - http://bmjopen.bmj.com/site/about/guidelines.xhtml

For peer review only

This was supported by patient responses, where they described the relationship they built with their physiotherapist.

> *For me, you know, having the same… the same desired outcome as the physiotherapist and* [wife] *you know, kind of, being all, all, all wanting the same thing. And it kind of felt if I did those things then I would eventually achieve it.* – QR08 (Age 43, Intervention arm participant)

> *she's brilliant, she's so lovely and fantastic hugger that's what I found if somebody you meet is happy to give you a hug when you are in this kind of situation it… it just makes everything so much easier… you* [physiotherapists] *not only do you do your jobs but when you're dealing with people like me you are counsellors as well* – QR12 (Age 55, Intervention arm participant)

> *she asked me how I felt and it was very much about me and my progress I had told her about how active my life was before my cancer and she was very supportive of… mmm… in terms of that so we want to get you back to we don't want anything, you know, we want you to get back to that… the goal… the ultimate goal so obviously without pushing yourself too hard we do want you to kind of challenge yourself in terms of trying to get that… to that end goal of you being able to do exercise* – QR13 (Age 28, Intervention arm participant)

Physiotherapists felt that having longer appointment times and an emphasis on shared goals and shared decisions, both of which encouraged exercise adherence, represented an ideal way of working. Many of the therapists remarked that they were pleased to be able to offer this service to breast cancer patients because of their previous experience of treating women struggling with chronic immobility, pain, and psychological issues in the longer term as a result of shoulder problems following breast cancer surgery.

> *When you pick up those patients* [later] *they come with a lot of emotional baggage and sort of their belief systems and it may have been years since they used their shoulder normally and then you know again if you've got body dysmorphic issues and they've been carrying that around for two years that's a lot more challenging to support.* – PT06 (Physiotherapist)

> *We get people coming in about two years later and they've never touched their scar, they never saw a physio, they're stiff, their scar's horrible, they've got awful myofascial trigger points and tightness…They still think two years down the line they're going to hurt themselves if they over stretch so if you get them in at the early stage then it's just better…I had a lady who had a mastectomy it was*

For peer review only - http://bmjopen.bmj.com/site/about/guidelines.xhtml

three years later she never went back to work, she never went back to any exercise, she never touched her scar, her mental wellbeing was like absolutely awful when I first started seeing her because she just didn't even know that she could have her life back. – PT01 (Physiotherapist)

I think we work too much reactive in the NHS don't we and I think a direction to move in is work in prevention rather than cure. – PT02 (Physiotherapist)

Physiotherapists connected this to the broader organisation of the NHS, and the need allocate resources to preventive care.

Delivering physiotherapy to breast cancer patients

This theme reports views on delivering a new physiotherapist-led intervention for breast cancer patients.

Meeting the needs of breast cancer patients

Participants and physiotherapists suggested that adjuvant treatment, such as chemotherapy, interfered with the patients' ability to maintain the exercise programme. After stopping the exercises when they felt unwell, it was physically more difficult to start doing the exercises again. Physiotherapists reflected that intervening at this point may have helped encourage and motivate patients to continue.

On day 17 after chemotherapy it has been a struggle…the last three weeks with the first lot of chemo this…[doing the exercises has] *been a lot harder than I ever anticipated.* – QR11 (Age 49, Intervention arm participant)

Maybe three or four times I was not well and I stopped doing the exercises for three or four days if I was ill and after that it was more difficult to do the exercises after. – QR10 (Age 50, Intervention arm participant)

A patient would come in for their first appointment and probably just post-surgery and most of them were quite positive had quite a lot of goals… they'd start their chemotherapy and then it was a whole different ball game because it was just kind of managing their fatigue and we struggled to get people back in for appointments. – PT02 (Physiotherapist)

For peer review only

The physiotherapists noted that patients needed emotional support, and that it was difficult to provide this in a curtained cubicle in an open-plan space, where patients potentially felt more vulnerable.

> *I think one of the things if I was to launch a service based on this intervention I would try and get a private treatment room 'cause we're working in curtained cubicles a lot of the time and I felt that didn't set the tone, I think if you're asking someone to take their bra off then and you can feel y'know curtains move with the best will in the world, not move open necessarily but you have that sense of 'Oh it's just a piece of material between me and God knows who'.* – PT10 (Physiotherapist)

Two therapists felt that physiotherapists should be female as they would better understand the meaning of losing a breast and more able to engage in the emotional and physical work of treating these patients.

> *They would probably connect better with a female and I was surprised how much women wanted to talk to me about their connection with their breasts so for a lot of them they felt like that was their femininity or that was um a connection to their womanhood and so I think most guys couldn't relate to how that feels so I could get where they were coming from.* – PT08 (Physiotherapist)

This issue was not mentioned by the trial participants we interviewed.

Emotional support for physiotherapists

Physiotherapists typically provide emotional support to patients, however, some therapists highlighted particular challenges in relation to this patient group due to the context of cancer treatment, for example, patients were fearful of dying from breast cancer. This was in contrast to their usual caseload which often involved caring for musculoskeletal patients with chronic conditions.

> *I am a person who cries quite easily so I was like 'Ok I need to keep things under control myself because I am the professional'… If I was to do it longer term I would need some better kind of guidance and help to deal with that…sometimes I felt a little bit lost.* – PT08 (Physiotherapist)

> *We were lucky because we had each other but there were times where it was upsetting to hear…If we were permanent members of staff in oncology you would be given some…de-briefing or kind of decompression but we were never offered that… both of us have had very close relatives die because of cancer…nobody considered that at all.* – PT06 (Physiotherapist)

For peer review only - http://bmjopen.bmj.com/site/about/guidelines.xhtml

For peer review only

The physiotherapists felt they would need emotional support if they worked routinely with breast cancer patients.

Physiotherapists' time, skills, and organisational integration

Delivering the intervention was time-consuming for physiotherapists.

> *I would say giving them the choice of exercise is time consuming which you wouldn't have in real life, you wouldn't have the time.* – PT09 (Physiotherapist)

Trial appointments were longer than usual and there were doubts about how this could be practically implemented as part of routine NHS clinical care given current time restrictions on appointments. The physiotherapists felt confident in identifying and treating physical shoulder problems, but often expressed a need for training about breast cancer, its treatments, and cancer specific complications. Cording, lymphedema, and seroma were unfamiliar postoperative complications to some physiotherapists until they took part in the trial.

> *We are MSK* [musculoskeletal] *physios and we know what a tight shoulder is and we know how to get it moving, so actually the assessment and the exercises wasn't so much of a worry, but patients occasionally asked me a question that maybe I couldn't answer…the background behind the cancer, a bit more about the actual surgical techniques they did and why and a little bit more about the reasoning of why lymphedema and cording does actually develop and what it means, I might have benefitted from more training from that aspect.* – PT03 (Physiotherapist)

> *Being able to advise people a bit more around like scar massage or kind of…any of the manual treatments that we could've done and when is right and wrong to use them* [was difficult]. – PT02 (Physiotherapist)

Physiotherapists felt disconnected from the surgical or oncology team treating the patient which was challenging.

> *There's not necessarily the integration with like the nurses or the lymphedema team, we are quite a separate team from them so I think it does need to be a multi-disciplinary approach and because we're not involved with them it makes it a little bit difficult* [to know] *whether we should or shouldn't be doing those interventions.* – PT03 (Physiotherapist)

> *I sometimes found it difficult to ask about things like chemo, radiotherapy and repeat surgeries because I almost felt like it was something that I should know… I find that, I feel a bit uncomfortable about that, that I think they come in, and expect, and that's what I'd want as a healthcare professional*

For peer review only - http://bmjopen.bmj.com/site/about/guidelines.xhtml

> *I want them to know what's going on I shouldn't have to tell you when I am having my chemo or this is happening.* – PT05 (Physiotherapist)

Better integration with the oncology team would have given them greater understanding of the specific patient's treatment schedule as they sometimes felt uncertain about whether the interventions were appropriate at a particular stage of cancer treatment.

Discussion

This qualitative study embedded within a large multicentre clinical trial makes a unique contribution to the literature. Our study illustrates that an individual supported exercise intervention is perceived as acceptable and beneficial by both patients and physiotherapists. Comparing the intervention and control arm enabled us to demonstrate that the intervention helped participants feel empowered and regaining a sense of control, whereas participants in the control arm spoke of passively accepting the upper limb limitations they experienced. Previous studies have explored perceptions of exercise in the context of an exercise intervention [36-38], but this is the first to include the perspectives of both intervention and control group participants, as well as physiotherapists delivering the intervention. We gained multiple perspectives on the same issue, and included all stakeholders in the study. This allowed us to triangulate and identify themes which were present across all groups. By using qualitative methods, we elicited the particular elements of the intervention which helped motivate participants, and those which were easier or more difficult to deliver in the clinical setting. This intervention is the first early structured physiotherapy-led home-based exercise intervention to be tested in breast cancer patients in the UK. An understanding of the acceptability of the intervention to patients and physiotherapists will inform future implementation strategies if the intervention is clinically and cost-effective.

Uncertainty has been identified as a feature of the experience of cancer [39, 29]. The subtheme of 'making progress' showed how witnessing improvement for themselves in terms of strength and stretching stood out in sharp contrast with the uncertainty surrounding cancer and its treatment. Participants also gained a sense of control over their progress, through being involved in choosing exercises, and through taking responsibility for completing their exercises each day (subthemes of 'helping myself' and looking ahead'). This combination appeared to restore patients' sense of autonomy over their bodies, and improved their wellbeing as they felt less

For peer review only - http://bmjopen.bmj.com/site/about/guidelines.xhtml

disempowered and hopeless. This echoes previous research which found feelings of increased empowerment when breast cancer patients participated in physical activity during active treatment [40-42]. These experiences contrasted to those in the control group, who did not experience the same sense of empowerment and progress. Specific aspects of the intervention which contributed to this sense of control over and above usual care were the contact with physiotherapists and the reassurance this provided, the sense of progress working through the prescribed programme as exercises increased in difficulty, and the shared decision-making used to select the home exercises. Previous research has found that participating in a group activity can be a way of forgetting about the illness [36]. Our study illustrates this can also be true for home-based or individually supported exercise programmes.

Being diagnosed with a serious illness such as cancer can cause an individual to lose trust and confidence in their bodily knowledge and of what their bodies are capable of doing [12, 43-45]. The women in this study reported kinesiophobia (fear of movement) in the acute period following surgery, but those in the intervention arm felt the intervention helped them overcome this (subtheme of 'reassurance'). Kinesiophobia has been shown to be associated with lymphoedema and greater pain intensity [33, 34]. Physiotherapists were able to reassure patients that their bodily sensations were normal, and gave them confidence to push themselves physically which motivated them to adhere to the programme. The interview data suggested that the role of the physiotherapist in affirming this progress and confidence was crucial. Physiotherapists provided invaluable emotional support, as patients unburdened onto them and shared their fears about the future and their bodies.

The physiotherapists enjoyed seeing positive improvements in the participants, and felt passionate about delivering what they viewed as high quality care to this patient group (subtheme of 'being the perfect therapist'). Physiotherapists felt satisfaction in being able to take preventive action against problems arising in the future for these women. Patients also appreciated the supportive nature of the intervention, sharing decisions and working together towards the same goal. Other authors have called for a more proactive model of health care provision for this patient group, and identified the need to improve physiotherapists' confidence in supporting these patients [46]. Our physiotherapists felt that they needed better integration with the rest of the patient's healthcare providers (final subtheme of 'Physiotherapists' time, skills, and organisational integration'). Other studies have also emphasised the importance of aligning expectations and knowledge about exercise based rehabilitation across the whole cancer care team [5] Challenges to the exercise programme were the side effects of treatment, in particular fatigue, which has been highlighted in other research as a barrier to exercise for breast cancer patients [36]. If a physiotherapist can provide motivation and encouragement during chemotherapy, this may improve adherence to

For peer review only - http://bmjopen.bmj.com/site/about/guidelines.xhtml

exercise. However, it is also important to ensure that physiotherapists are sympathetic to treatment-related issues, and can tailor programmes during these periods of fatigue [36].

The theme 'Delivering physiotherapy to breast cancer patients' highlighted considerations for the implementation of an exercise intervention for breast cancer patients. The intervention should be delivered in a private walled room, ideally with a specifically-trained (female) physiotherapist who is part of the multi-disciplinary oncology team caring for the patient. The most important ingredient of the intervention was contact with the physiotherapists, suggesting resources should be focused on training and supporting physiotherapists to provide this care. Some physiotherapists reported feeling upset when treating patients because of the patients' distress or their own experiences of cancer. This suggests that healthcare professionals caring for oncology patients should be given the opportunity of debriefing and emotional support. This is an important consideration when designing future interventions for this patient group.

In the PROSPER trial, participants underwent a one hour assessment and then subsequent 30-minute follow-up appointments. Routine UK physiotherapy outpatient appointments are often 40 minutes (assessment) and 20 minutes (follow-up). Physiotherapists worked with patients to select the exercises. This may be challenging to deliver in a resource-stretched NHS context. However, longer appointments with physiotherapists, creating shared goals, and making shared decisions about exercises, were viewed as the most important ingredients in the successful delivery of the intervention. This was brought out in all our themes. Other studies have highlighted how autonomy of choice over exercises may increase motivation and adherence [37, 47]. Additionally, we provided the PROSPER materials in an attractive ring binder with colour photographs, laminated sheets, and provided exercise diary handouts. Patient-participants said the diary was useful as a prompt to remember to do their exercises, and it was helpful to see photographs of the exercises.

Acting more proactively by providing good access to physiotherapy treatment early after, or alongside, breast cancer treatment could help to reduce the number of patients with cancer presenting with musculoskeletal complications [48]. Although our physiotherapist-participants felt very comfortable with aspects of the intervention such as improving shoulder mobility, they expressed a need for greater training, support, and guidance in relation to specific issues such as cording and lymphoedema. The physiotherapists delivering the PROSPER intervention were musculoskeletal specialists with limited experience in treating patients in the acute postoperative period. Physiotherapists in the UK receive little training in rehabilitation following cancer treatment, reflected by the limited centres across the UK with physiotherapists specialised in oncology [48]. Given the

For peer review only - http://bmjopen.bmj.com/site/about/guidelines.xhtml

increasing number of people surviving cancer and living with the consequences of cancer treatment, there is an urgent need in the UK to upskill physiotherapists in cancer-related rehabilitation to allow patients better access to this type of rehabilitation.

Conclusion

This study has highlighted how a physiotherapist-led home exercise programme, with built-in progression and shared decision-making, helped women undergoing breast cancer treatment gain a restored sense of control over their wellbeing, and empowered them during a highly disempowering experience.

Author statement

JB is Chief Investigator of the PROSPER Trial. JB/SR/EW/HR/BM contributed to the study protocol. SR collected and analysed the data. JB/BM/HR/JB/EW assisted with analysis and interpretation. SR led the writing of the paper, and all other authors contributed to writing and editing.

Funding

Funding for the PROSPER trial was provided by the Health Technology Assessment programme of the National Institute for Health Research (NIHR) (Project Number 13/84/10). JB is supported by NIHR Research Capability Funding via University Hospitals Coventry and Warwickshire. EW is supported by the NIHR Applied Research Collaboration Oxford and Thames Valley.

Conflicts of interest

The authors have no conflicts of interest.

For peer review only

Data availability statement

The datasets generated during and/or analysed during the current study are not publicly available due to the need to protect the identity of participants.

Patient and Public Involvement

Patients partnered with us for the design of the study, the informational material to support the qualitative research, and the burden of the interview from the patient's perspective.

Acknowledgements

We would like to thank all the physiotherapists and trial participants who took part in this interview study. We dedicate this paper to Elizabeth (Lizzie) Abbey (nee Fort) who passed away on 3rd April 2018. Lizzie helped set up the trial and was a highly valued member of the PROSPER team.

References

1. McNeely ML, Campbell K, Ospina M, Rowe BH, Dabbs K, Klassen TP et al. Exercise interventions for upper-limb dysfunction due to breast cancer treatment. Cochrane Database Syst Rev. 2010(6). doi:10.1002/14651858.CD005211.pub2.
2. Mejdahl MK, Andersen KG, Gärtner R, Kroman N, Kehlet H. Persistent pain and sensory disturbances after treatment for breast cancer: six year nationwide follow-up study. BMJ. 2013;346:f1865.
3. Cardoso F, Kyriakides S, Ohno S, Penault-Llorca F, Poortmans P, Rubio IT et al. Early breast cancer: ESMO Clinical Practice Guidelines for diagnosis, treatment and follow-up. Ann Oncol. 2019. doi:10.1093/annonc/mdz173.
4. Excellence NIfC. Early and locally advanced breast cancer: diagnosis and management NICE guideline [NG101]. 2018. https://www.nice.org.uk/guidance/ng101/chapter/Recommendations. Accessed 31st January 2020.
5. Dennett AM, Harding KE, Reed MS. The challenge of timing: a qualitative study on clinician and patient perspectives about implementing exercise-based rehabilitation in an acute cancer treatment setting. Support Care Cancer. 2020. doi:10.1007/s00520-020-05436-7.
6. Fisher MI, Howell D. The power of empowerment: An ICF-based model to improve self-efficacy and upper extremity function of survivors of breast cancer. Rehabil Oncol. 2010;28(3).
7. Dunn J, Steginga SK. Young women's experience of breast cancer: defining young and identifying concerns. Psychooncology. 2000;9(2):137-46.

For peer review only - http://bmjopen.bmj.com/site/about/guidelines.xhtml

8. Thomas-MacLean R. Memories of treatment: the immediacy of breast cancer. Qual Health Res. 2004;14(5):628-43. doi:10.1177/1049732304263658.
9. Little M, Jordens CF, Paul K, Montgomery K, Philipson B. Liminality: a major category of the experience of cancer illness. Soc Sci Med. 1998;47(10):1485-94.
10. McCann L, Illingworth N, Wengström Y, Hubbard G, Kearney N. Transitional experiences of women with breast cancer within the first year following diagnosis. J Clin Nurs. 2010;19(13-14):1969-76.
11. Trusson D, Pilnick A, Roy S. A new normal?: Women's experiences of biographical disruption and liminality following treatment for early stage breast cancer. Soc Sci Med. 2016.
12. Rees S. No one scans you and says 'you're alright now': the experience of embodied risk for young women living with a history of breast cancer. Health, Risk & Society. 2018:1-13. doi:10.1080/13698575.2018.1539468.
13. Kim S, Han J, Lee MY, Jang MK. The experience of cancer-related fatigue, exercise and exercise adherence among women breast cancer survivors: Insights from focus group interviews. J Clin Nurs. 2020;29(5-6):758-69. doi:10.1111/jocn.15114.
14. Livsey L, Lewis K. Breast cancer survivors' perceptions of participating in a supervised exercise intervention: An exploratory review of the literature. Women Health. 2018;58(9):1017-36. doi:10.1080/03630242.2017.1372844.
15. Kenyon K Mres PT, Hebron C PhD PT, Vuoskoski P PhD PT, McCrum C Dprof PT. Physiotherapists' experiences of managing upper limb movement impairments due to breast cancer treatment. Physiother Theory Pract. 2018:1-14. doi:10.1080/09593985.2018.1480077.
16. Bruce J, Williamson E, Lait C, Richmond H, Betteley L, Lall R et al. Randomised controlled trial of exercise to prevent shoulder problems in women undergoing breast cancer treatment: study protocol for the prevention of shoulder problems trial (UK PROSPER). BMJ Open. 2018;8(3):e019078. doi:10.1136/bmjopen-2017-019078.
17. Richmond H, Lait C, Srikesavan C, Williamson E, Moser J, Newman M et al. Development of an exercise intervention for the prevention of musculoskeletal shoulder problems after breast cancer treatment: the prevention of shoulder problems trial (UK PROSPER). BMC Health Serv Res. 2018;18(1):463. doi:10.1186/s12913-018-3280-x.
18. Archer MS. Realist social theory: The morphogenetic approach. Cambridge University Press; 1995.
19. Bhaskar R. A realist theory of science. Routledge; 2013.
20. Braun V, Clarke V. Reflecting on reflexive thematic analysis. Qualitative Research in Sport, Exercise and Health. 2019;11(4):589-97. doi:10.1080/2159676X.2019.1628806.
21. O'Brien BC, Harris IB, Beckman TJ, Reed DA, Cook DAJAM. Standards for reporting qualitative research: a synthesis of recommendations. 2014;89(9):1245-51.
22. Ltd QIP. NVivo qualitative data analysis software. QSR International Pty Ltd. Stokholm, Sweden; 2008.
23. Guest G, MacQueen KM, Namey EE. Applied thematic analysis. Sage; 2011.
24. Boyatzis RE. Transforming qualitative information: Thematic analysis and code development. sage; 1998.
25. Charmaz K. Constructing grounded theory. Sage; 2014.
26. Braun V, Clarke V. To saturate or not to saturate? Questioning data saturation as a useful concept for thematic analysis and sample-size rationales. Qualitative Research in Sport, Exercise and Health. 2019:1-16. doi:10.1080/2159676X.2019.1704846.
27. Hennink MM, Kaiser BN, Marconi VC. Code saturation versus meaning saturation: how many interviews are enough? Qual Health Res. 2017;27(4):591-608.
28. Rees S. A qualitative exploration of the meaning of the term "survivor" to young women living with a history of breast cancer. European journal of cancer care. 2018;27(3):e12847.
29. Rees S. 'Am I really gonna go sixty years without getting cancer again?'Uncertainty and liminality in young women's accounts of living with a history of breast cancer. Health:. 2017;21(3):241–58. doi:10.1177/1363459316677628.
30. Rees S, Young A. The experiences and perceptions of women diagnosed with breast cancer during pregnancy. Asia-Pacific journal of oncology nursing. 2016;3(3):252-8. doi:10.4103/2347-5625.189814.
31. Hutchinson A, Rees S, Young A, Maraveyas A, Date K, Johnson MJ. Oral anticoagulation is preferable to injected, but only if it is safe and effective: An interview study of patient and carer experience of oral and injected anticoagulant therapy for cancer-associated thrombosis in the select-d trial. Palliat Med. 2019;33(5):510-7.
32. Lincoln YS, Guba EG. But is it rigorous? Trustworthiness and authenticity in naturalistic evaluation. New directions for program evaluation. 1986;1986(30):73-84.
33. Gencay Can A, Can SS, Ekşioğlu E, Çakcı FA. Is kinesiophobia associated with lymphedema, upper extremity function, and psychological morbidity in breast cancer survivors? Turkish journal of physical medicine and rehabilitation. 2018;65(2):139-46. doi:10.5606/tftrd.2019.2585.

For peer review only

For peer review only - http://bmjopen.bmj.com/site/about/guidelines.xhtml

34. Martinez-Calderon J, Struyf F, Meeus M, Luque-Suarez A. The association between pain beliefs and pain intensity and/or disability in people with shoulder pain: A systematic review. Musculoskelet Sci Pract. 2018;37:29-57. doi:10.1016/j.msksp.2018.06.010.
35. Trusson D. Living with a new normal: women's experiences following treatment for early-stage breast cancer or DCIS: University of Nottingham; 2013.
36. Browall M, Mijwel S, Rundqvist H, Wengstrom Y. Physical Activity During and After Adjuvant Treatment for Breast Cancer: An Integrative Review of Women's Experiences. Integr Cancer Ther. 2018;17(1):16-30. doi:10.1177/1534735416683807.
37. Ingram C, Wessel J, Courneya KS. Women's perceptions of home-based exercise performed during adjuvant chemotherapy for breast cancer. Eur J Oncol Nurs. 2010;14(3):238-43. doi:10.1016/j.ejon.2010.01.027.
38. Luoma ML, Hakamies-Blomqvist L, Blomqvist C, Nikander R, Gustavsson-Lilius M, Saarto T. Experiences of breast cancer survivors participating in a tailored exercise intervention -a qualitative study. Anticancer Res. 2014;34(3):1193-9.
39. Halliday LE, Boughton MA, Kerridge I. Mothering and self-othering: The impact of uncertain reproductive capability in young women after hematological malignancy. Health Care Women Int. 2014;35(3):249-65.
40. Husebo AM, Karlsen B, Allan H, Soreide JA, Bru E. Factors perceived to influence exercise adherence in women with breast cancer participating in an exercise programme during adjuvant chemotherapy: a focus group study. J Clin Nurs. 2015;24(3-4):500-10. doi:10.1111/jocn.12633.
41. Crane-Okada R, Kiger H, Anderson NL, Carroll-Johnson RM, Sugerman F, Shapiro SL et al. Participant perceptions of a mindful movement program for older women with breast cancer: focus group results. Cancer Nurs. 2012;35(3):E1-10. doi:10.1097/NCC.0b013e31822539c5.
42. Bulmer SM, Howell J, Ackerman L, Fedric R. Women's perceived benefits of exercise during and after breast cancer treatment. Women Health. 2012;52(8):771-87. doi:10.1080/03630242.2012.725707.
43. Williams SJ. The vicissitudes of embodiment across the chronic illness trajectory. Body & Society. 1996;2(2):23-47.
44. Lindwall L, Bergbom I. The altered body after breast cancer surgery. International Journal of Qualitative Studies on Health and Well-being. 2009;4(4):280-7.
45. Crouch M, McKenzie H. Social realities of loss and suffering following mastectomy. Health:. 2000;4(2):196-215.
46. Levangie PK, Santasier AM, Stout NL, Pfalzer L. A qualitative assessment of upper quarter dysfunction reported by physical therapists treated for breast cancer or treating breast cancer sequelae. Support Care Cancer. 2011;19(9):1367-78. doi:10.1007/s00520-010-0959-x.
47. Jones LW, Courneya KS, Fairey AS, Mackey JR. Does the theory of planned behavior mediate the effects of an oncologist's recommendation to exercise in newly diagnosed breast cancer survivors? Results from a randomized controlled trial. Health Psychol. 2005;24(2):189.
48. Robinson M, Ward L, Mehanna H, Paleri V, Winter SC. Provision of physiotherapy rehabilitation following neck dissection in the UK. The Journal of Laryngology & Otology. 2018;132(7):624-7. doi:10.1017/S0022215118000671.

For peer review only - http://bmjopen.bmj.com/site/about/guidelines.xhtml

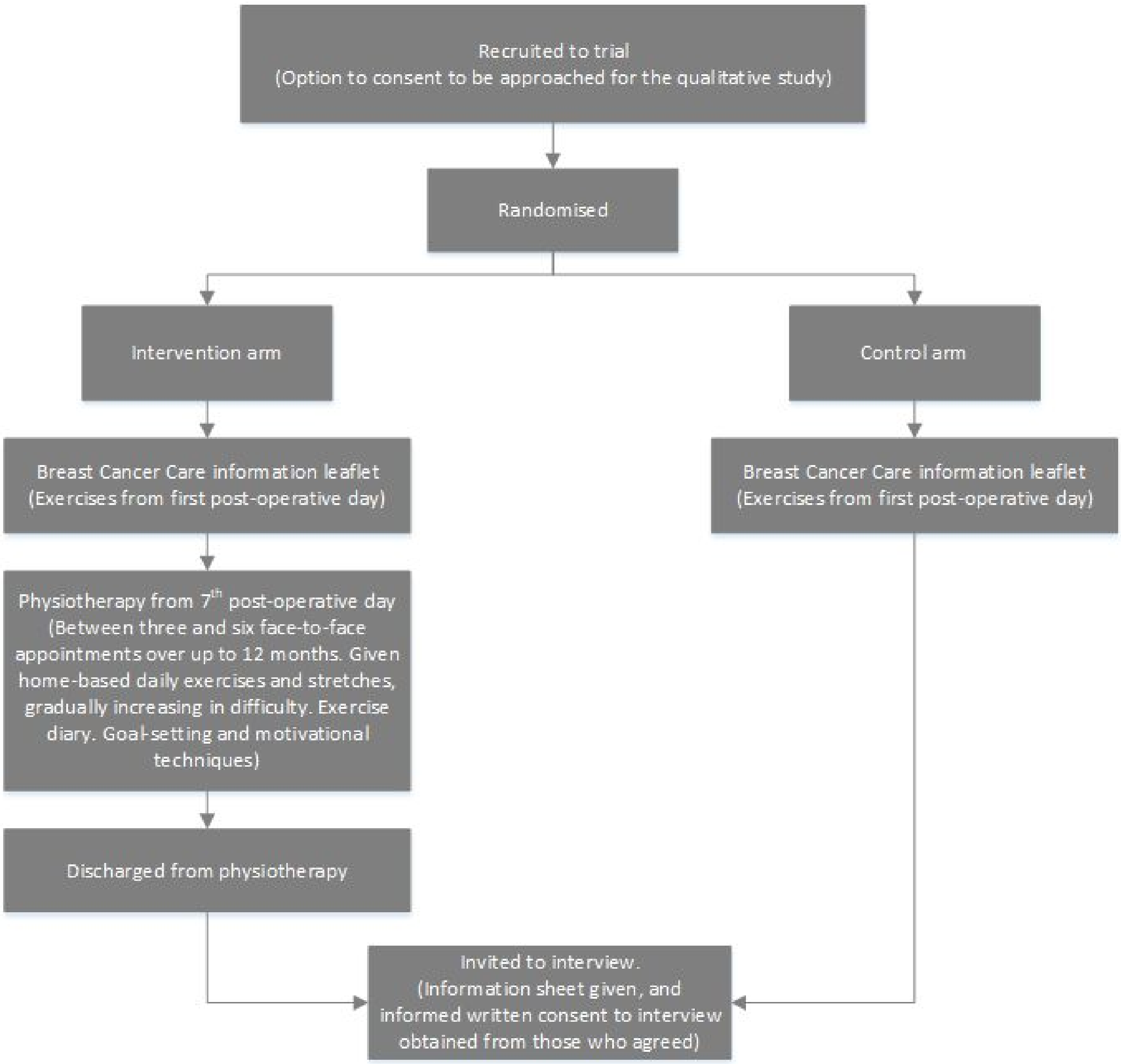

Figure 1- Participant pathway through trial and qualitative study

Reporting checklist for qualitative study.

Based on the SRQR guidelines.

Instructions to authors

Complete this checklist by entering the page numbers from your manuscript where readers will find each of the items listed below.

Your article may not currently address all the items on the checklist. Please modify your text to include the missing information. If you are certain that an item does not apply, please write "n/a" and provide a short explanation.

Upload your completed checklist as an extra file when you submit to a journal.

In your methods section, say that you used the SRQRreporting guidelines, and cite them as:

O'Brien BC, Harris IB, Beckman TJ, Reed DA, Cook DA. Standards for reporting qualitative research: a synthesis of recommendations. Acad Med. 2014;89(9):1245-1251.

		Reporting Item	Page Number
**Title**			
	#1	Concise description of the nature and topic of the study identifying the study as qualitative or indicating the approach (e.g. ethnography, grounded theory) or data collection methods (e.g. interview, focus group) is recommended	
**Abstract**			
	#2	Summary of the key elements of the study using the abstract format of the intended publication; typically includes background, purpose, methods, results and conclusions	
**Introduction**			
Problem formulation	#3	Description and signifcance of the problem / phenomenon studied: review of relevant theory and empirical work; problem statement	
Purpose or research question	#4	Purpose of the study and specific objectives or questions	

For peer review only

Methods

Qualitative approach and research paradigm	#5	Qualitative approach (e.g. ethnography, grounded theory, case study, phenomenolgy, narrative research) and guiding theory if appropriate; identifying the research paradigm (e.g. postpositivist, constructivist / interpretivist) is also recommended; rationale. The rationale should briefly discuss the justification for choosing that theory, approach, method or technique rather than other options available; the assumptions and limitations implicit in those choices and how those choices influence study conclusions and transferability. As appropriate the rationale for several items might be discussed together.
Researcher characteristics and reflexivity	#6	Researchers' characteristics that may influence the research, including personal attributes, qualifications / experience, relationship with participants, assumptions and / or presuppositions; potential or actual interaction between researchers' characteristics and the research questions, approach, methods, results and / or transferability
Context	#7	Setting / site and salient contextual factors; rationale
Sampling strategy	#8	How and why research participants, documents, or events were selected; criteria for deciding when no further sampling was necessary (e.g. sampling saturation); rationale
Ethical issues pertaining to human subjects	#9	Documentation of approval by an appropriate ethics review board and participant consent, or explanation for lack thereof; other confidentiality and data security issues
Data collection methods	#10	Types of data collected; details of data collection procedures including (as appropriate) start and stop dates of data collection and analysis, iterative process, triangulation of sources / methods, and modification of procedures in response to evolving study findings; rationale
Data collection instruments and technologies	#11	Description of instruments (e.g. interview guides, questionnaires) and devices (e.g. audio recorders) used for data collection; if / how the instruments(s) changed over the course of the study
Units of study	#12	Number and relevant characteristics of participants, documents, or events included in the study; level of participation (could be

For peer review only

		reported in results)
Data processing	#13	Methods for processing data prior to and during analysis, including transcription, data entry, data management and security, verification of data integrity, data coding, and anonymisation / deidentification of excerpts
Data analysis	#14	Process by which inferences, themes, etc. were identified and developed, including the researchers involved in data analysis; usually references a specific paradigm or approach; rationale
Techniques to enhance trustworthiness	#15	Techniques to enhance trustworthiness and credibility of data analysis (e.g. member checking, audit trail, triangulation); rationale
**Results/findings**		
Syntheses and interpretation	#16	Main findings (e.g. interpretations, inferences, and themes); might include development of a theory or model, or integration with prior research or theory
Links to empirical data	#17	Evidence (e.g. quotes, field notes, text excerpts, photographs) to substantiate analytic findings
**Discussion**		
Intergration with prior work, implications, transferability and contribution(s) to the field	#18	Short summary of main findings; explanation of how findings and conclusions connect to, support, elaborate on, or challenge conclusions of earlier scholarship; discussion of scope of application / generalizability; identification of unique contributions(s) to scholarship in a discipline or field
Limitations	#19	Trustworthiness and limitations of findings
**Other**		
Conflicts of interest	#20	Potential sources of influence of perceived influence on study conduct and conclusions; how these were managed
Funding	#21	Sources of funding and other support; role of funders in data collection, interpretation and reporting

None The SRQR checklist is distributed with permission of Wolters Kluwer © 2014 by the Association of American Medical Colleges. This checklist can be completed online using https://www.goodreports.org/, a tool made by the EQUATOR Network in collaboration with Penelope.ai

For peer review only

BMJ Open

The role of physiotherapy in supporting recovery from breast cancer treatment: A qualitative study embedded within the UK PROSPER trial

Journal:	*BMJ Open*
Manuscript ID	bmjopen-2020-040116.R2
Article Type:	Original research
Date Submitted by the Author:	18-Nov-2020
Complete List of Authors:	Rees, Sophie; University of Warwick, Warwick Clinical Trials Unit<br>Mazuquin, Bruno; University of Warwick, Warwick Clinical Trials Unit; Manchester Metropolitan University, Faculty of Health, Psychology and Social Care<br>Richmond, Helen ; University of Warwick, Warwick Clinical Trials Unit; Memorial University of Newfoundland, Faculty of Medicine<br>Williamson, Esther; Nuffield Department of Orthopaedics, Rheumatology and Musculoskeletal Sciences, University of Oxford, United Kingdom. , Centre for Rehabilitation Research in Oxford<br>Bruce, Julie; University of Warwick, Warwick Clinical Trials Unit<br>-, UK PROSPER Study Group; University of Warwick, Warwick Clinical Trials Unit
**Primary Subject Heading**:	Oncology
Secondary Subject Heading:	Qualitative research, Surgery
Keywords:	QUALITATIVE RESEARCH, Breast surgery < SURGERY, PREVENTIVE MEDICINE

SCHOLARONE™ Manuscripts

I, the Submitting Author has the right to grant and does grant on behalf of all authors of the Work (as defined in the below author licence), an exclusive licence and/or a non-exclusive licence for contributions from authors who are: i) UK Crown employees; ii) where BMJ has agreed a CC-BY licence shall apply, and/or iii) in accordance with the terms applicable for US Federal Government officers or employees acting as part of their official duties; on a worldwide, perpetual, irrevocable, royalty-free basis to BMJ Publishing Group Ltd ("BMJ") its licensees and where the relevant Journal is co-owned by BMJ to the co-owners of the Journal, to publish the Work in this journal and any other BMJ products and to exploit all rights, as set out in our licence.

The Submitting Author accepts and understands that any supply made under these terms is made by BMJ to the Submitting Author unless you are acting as an employee on behalf of your employer or a postgraduate student of an affiliated institution which is paying any applicable article publishing charge ("APC") for Open Access articles. Where the Submitting Author wishes to make the Work available on an Open Access basis (and intends to pay the relevant APC), the terms of reuse of such Open Access shall be governed by a Creative Commons licence – details of these licences and which Creative Commons licence will apply to this Work are set out in our licence referred to above.

Other than as permitted in any relevant BMJ Author's Self Archiving Policies, I confirm this Work has not been accepted for publication elsewhere, is not being considered for publication elsewhere and does not duplicate material already published. I confirm all authors consent to publication of this Work and authorise the granting of this licence.

For peer review only

The role of physiotherapy in supporting recovery from breast cancer treatment: A qualitative study embedded within the UK PROSPER trial

Rees S,[1] Mazuquin B,[1,2] Richmond H,[1,3] Williamson E,[4] Bruce J.[1] on behalf of the UK PROSPER Study Group[1]

[1]*Warwick Clinical Trials Unit, University of Warwick Medical School, Coventry, UK CV4 7AL*

[2] *Faculty of Health, Psychology and Social Care, Manchester Metropolitan University*

[3]*Faculty of Medicine, Memorial University of Newfoundland, St John's, Canada*

[4]*The Centre for Rehabilitation Research, Nuffield Department of Orthopaedics, Rheumatology and Musculoskeletal Sciences, University of Oxford*

Corresponding author:
Dr Sophie Rees
S.Rees.1@warwick.ac.uk
02476 151 135

For peer review only

Abstract

Objectives: To explore the experiences of women with breast cancer of taking part in an early physiotherapy-led exercise intervention compared with the experiences of those receiving usual care. To understand physiotherapists' experience of delivering the trial intervention. To explore acceptability of the intervention and issues related to the implementation of the PROSPER programme from participant and physiotherapist perspective.

Design: Qualitative semi-structured interviews with thematic analysis.

Setting: UK National Health Service (NHS)

Participants: Twenty participants at high risk of shoulder problems after breast cancer surgery, recruited to the UK Prevention of Shoulder Problems (PROSPER) Trial (ten each from the intervention arm and control arm); and eleven physiotherapists who delivered the intervention. Trial participants were sampled using convenience sampling. Physiotherapists were purposively sampled from high and low recruiting sites.

Results: Participants described that the PROSPER exercise intervention helped them feel confident in what their body could do, and helped them regain a sense of control in the context of cancer treatment which was largely

For peer review only - http://bmjopen.bmj.com/site/about/guidelines.xhtml

disempowering. Control arm participants expressed less of a sense of control over their wellbeing. Physiotherapists found the exercise intervention enjoyable to deliver and felt it was valuable to their patients. The extra time allocated for appointments during intervention delivery made physiotherapists feel they were providing optimal care, being the 'perfect physio'. Lessons were learned about the implementation of a complex exercise intervention for women with breast cancer and the issues raised will inform the development of a future implementation strategy.

Conclusions: A physiotherapist-delivered early supported exercise intervention with integrated behavioural strategies helped women at risk of shoulder problems following breast cancer treatment to feel more confident in their ability to mobilise their arm post-surgery. A physiotherapist-delivered early supported exercise intervention with integrated behavioural strategies may address the sense of powerlessness that many women experience during breast cancer treatment.

Keywords: *breast cancer; physiotherapy; rehabilitation; prevention; shoulder; qualitative research*

Acknowledgements

This study was embedded within a multicentre UK randomised controlled trial (ISCRTN35358984) and was funded by the National Institute for Health Research Health Technology Assessment Programme (HTA 13/84/10). The research team would like to thank the trial participants and physiotherapists who participated in the qualitative study.

Word count: 6,259 (due to quotes being included in word count)

For peer review only - http://bmjopen.bmj.com/site/about/guidelines.xhtml

Article Summary

Strengths and limitations of this study

- Interviewing multiple groups (intervention arm, control arm participants, and physiotherapists) in this study enabled us to triangulate the data and explore experiences from multiple perspectives.
- We note that the participants we interviewed were a particularly motivated group, and it is possible we did not capture some of the challenges which other, less motivated, women may have experienced.
- We obtained consent to be approached for interview prior to randomisation, independent of treatment allocation, in an attempt to minimise bias. We tried to minimise the risk of social desirability bias by asking neutral questions and explaining there were no right or wrong answers
- We used a convenience sampling approach, which is a potential weakness of this study as it may have resulted in a lack of diversity amongst participants.
- Our sample was overwhelmingly white, with only one of the participants identifying as another ethnic identity. Findings may not reflect the experiences of black, Asian and other minority ethnic groups.

Introduction

Treatment to the chest and axilla for breast cancer can result in upper body problems, such as reduced range of movement in the shoulder, muscle weakness, pain, lymphoedema, and functional limitations [1, 2]. These problems can impact on ability to carry out activities of daily living, and may persist for many years after treatment [1, 2]. Exercise in the acute phase following breast cancer surgery may improve shoulder function in women at high risk of shoulder problems [1]. Guidelines state that people diagnosed with breast cancer should be referred to physiotherapy when indicated [3, 4], however, in the UK NHS this is not routine practice. There is a need for a proactive model of care which encourages early exercise-based rehabilitation and provides physiotherapists with resources to inform their practice [5].

Loss of a sense of control, loss of self-identity, and alienation from their bodies during and after treatment are often reported by individuals with cancer [6-12]. It has been proposed that improving women's self-efficacy through physical rehabilitation may improve their quality of life [6]. Lack of knowledge about exercise, and the

For peer review only - http://bmjopen.bmj.com/site/about/guidelines.xhtml

experience of cancer-related fatigue were identified by individuals with cancer as obstacles to exercise in a recent Korean study [13]. A recent systematic review of the qualitative literature identified six studies of mixed quality reporting the experiences of women living beyond breast cancer of participating in a supervised exercise intervention [14]. These studies all reported on group interventions, and the findings suggest that the group element may be beneficial. Little is known about the experiences of this patient group participating in individual supported exercise intervention. There has also been little published regarding the experiences of professionals delivering exercise interventions to this patient group. One recent study reported a lack of confidence amongst physiotherapists in treating people with cancer, but respondents felt confidence grew with practice [15]. Little is known about how physiotherapists feel about the feasibility of implementing a service for people with breast cancer. This is important so that we can address challenges and issues when designing services.

The UK Prevention Of Shoulder Problems (PROSPER) Trial evaluated the clinical and cost-effectiveness of an early supported home-based physiotherapist-led exercise intervention in women with newly diagnosed breast cancer at higher risk of developing shoulder problems after treatment [16, 17]. We have published a description of the intervention and trial protocol elsewhere [16, 17]. In this paper we report the findings of the UK PROSPER trial embedded qualitative study.

The aims of the qualitative study were:

- To understand the acceptability of the exercise intervention to participants
- To explore how the exercise intervention or control affected their experiences of recovery after cancer treatment.
- To investigate the experiences of physiotherapists delivering the exercise intervention.
- To explore participants' and physiotherapists' perspectives on issues related to the implementation of the PROSPER programme to inform future plans for implementation. .

Figure 1 illustrates the pathway of participants through the trial and embedded qualitative study.

Figure 1 - Participant pathway through trial and qualitative study

For peer review only

For peer review only - http://bmjopen.bmj.com/site/about/guidelines.xhtml

Methods

Methodology

The study was underpinned by critical realism, assuming that an underlying reality is experienced and given meaning by individuals [18, 19]. To meet the study aims, we conducted qualitative semi-structured interviews with reflexive thematic analysis [20]. This allowed for exploration, depth, and understanding of the experiences of trial participants, thus taking an interpretive 'sense-making' approach rather than hypothesis-testing or confirmatory approach. We used the SRQR reporting guidelines checklist [21].

Sampling and recruitment

Trial participant interviews

On recruitment to the trial, we offered all trial participants the option to take part in an interview at a later date (see Figure 1). We recorded signed consent to be approached for interview and this formed our sampling frame for the qualitative study. We approached women in the intervention arm after they were discharged from physiotherapy to avoid contamination bias. The researcher (SR) telephoned participants to invite them to interview, and, if they expressed an interest, participants were sent an information sheet and interview consent form.

After conducting and analysing seven interviews with intervention participants, we decided to interview control arm participants, to compare their experiences. We used our database of those who had consented pre-randomisation to select a sample comparable to the intervention sample in terms of time since randomisation, so that women were at similar stages of postoperative treatment and could reflect back over their experiences of recovery.

Physiotherapist interviews

We informed all physiotherapists delivering the intervention about the interview study. We then sampled physiotherapists from low and high recruiting trial sites to allow exploration of different perspectives on intervention delivery. SR approached therapists via email or telephone.

For peer review only - http://bmjopen.bmj.com/site/about/guidelines.xhtml

Data collection

We developed flexible topic guides with women with breast cancer, based on the aims of the study and relevant literature. One-off interviews were conducted by SR either at the participant's home, by telephone (trial participants), or in a private room at their place of work (physiotherapists). Physiotherapists who worked together were interviewed in pairs. The physiotherapists who volunteered for the interviews and were interviewed in pairs worked closely together. Interviewing them in pairs allowed physiotherapists to share and reflect on their experiences, and aided recall where they had only treated a small number of participants, for example. It is possible that interviewing them in pairs could have affected their responses, but participants were remarkably candid about the challenges they experienced, thus we were not concerned that this was happening. Only the researcher and interviewees were present. All interviews were audio-recorded. We took study materials (physiotherapy manual, participant materials) into the interview to aid recall and discussion. Informed consent was gained before the interview began. The study was approved by the National Research Ethics Service Committee West Midlands Solihull on 20th July 2015 (Ref no. 15/WM/0224),

Data analysis

Interviews were transcribed verbatim, checked for accuracy and anonymity by SR, and then uploaded to QSR NVivo Pro 11 [22]. Thematic analysis [23, 24] was conducted by SR and managed in NVivo. Interview transcripts were 'coded', where sections of text are assigned a descriptive label, producing dozens of codes per interview. These codes were then grouped into categories, and these were then grouped further into themes. Analysis began alongside data collection. As a research team, we met regularly to discuss emerging findings and the evolving analysis [25]. Saturation in this study meant that we had enough data to understand each of the identified categories and themes, rather than that there was 'nothing new' to be found [26, 27]. We reached saturation after fifteen trial participant interviews, and five physiotherapist interviews.

Reflexivity and rigour

Interviews were conducted sensitively by a female researcher experienced in interviewing people with cancer (SR) [12, 28-31]. The evolving analysis was discussed with the research team (SR/JB/HR/BM). SR is a social scientist with expertise in qualitative research with people with health conditions, including breast cancer, and healthcare professionals. HR and BM are researchers and physiotherapists. JB is a trialist and PROSPER Chief

For peer review only - http://bmjopen.bmj.com/site/about/guidelines.xhtml

Investigator, she did not influence the qualitative study findings, but provided important contextual details regarding the trial and intervention. We were careful to conduct balanced interviews, without assuming that the trial participants and physiotherapists would have positive views of the intervention. SR reminded interviewees throughout that she was not involved in the development of the intervention, and welcomed their honest views. Rigour was assessed using Lincoln and Guba's conceptualisation of trustworthiness [32]. SR collected the data and was immersed in the data during analysis. Quotes have been provided to illustrate themes.

Results

Sample

We recruited 392 women (196 per arm) to the PROSPER clinical trial from 17 breast cancer centres in England. Overall, 67% (n=264/392) of trial participants provided signed consent to be contacted for an interview. In total, we attempted to contact 53 women regarding an interview. Of these, 11 were not contactable, 17 agreed initially for an interview but could not be reached again, and five declined. Ten participants from the intervention arm and ten from the control arm were interviewed from 11 of the 17 study sites (see Table 1). There were no apparent differences between the sites regarding the issues raised by trial participants and therapists. We had a good range in terms of size and rural/urban sites across the 11 sites represented in the interview study.

Interviews were carried out with 11/44 (25%) physiotherapists (all female) from six study sites. Ten were interviewed in pairs and one individually. The physiotherapists had treated between one and 16 trial participants (median 5) and were based at hospitals that did not routinely provide postoperative physiotherapy after breast cancer surgery. They were experienced in the management of musculoskeletal conditions but did not currently work in breast cancer or oncology units. Some physiotherapists had experience of treating people with breast cancer presenting with problems such as restricted shoulder movement preventing the start of radiotherapy. One physiotherapist had past experience working on a cancer inpatient ward.

Table 1 - Study sample (trial participants)

**Characteristic**	**Intervention arm N=10**	**Control arm N=10**
Months since randomisation, mean*(range)*	7 (3-11)	7 (3-12)
Age at randomisation, mean *(range)*	51 (28-69)	60 (44-79)

For peer review only - http://bmjopen.bmj.com/site/about/guidelines.xhtml

For peer review only

Age at randomisation		
18-29	1	0
30-39	0	0
40-49	4	2
50-59	3	3
60-69	2	3
70-79	0	2
Ethnicity		
White	9	10
Mixed	1	0
Surgical treatment*		
Mastectomy	4	3
Breast conserving surgery	6	7
Axillary node clearance	10	8
Sentinel lymph node biopsy	3	4
Adjuvant therapy*		
Chemotherapy	9	7
Radiotherapy	9	10

**participants had multiple treatments*

We identified three themes from the data: 'healing'; 'being the perfect therapist'; and 'delivering physiotherapy to women with breast cancer'. The themes and subthemes are illustrated by participant group in Table 2, and each theme is described below with subthemes.

For peer review only

Table 2 - Illustration of themes and subthemes by participant group

Theme	Subtheme	Intervention group	Control group	Physiotherapists
Healing	Reassurance	*The physiotherapist was able to tell you whether you were doing things right or wrong*	*It's quite tender…you don't feel like you ought to be doing it…you feel like it's too soon*	*They think they're going to split their stitches…it's just the reassurance we give*
	Making progress	*You saw results and sometimes with your cancer…you don't see results until the end*	*I'm tender but I suppose that will go*	
	Helping myself	*I think it was because you were doing something, because so much of cancer care is being done to you*	*It still gets stiff now but you just have to deal with it.*	*They had the confidence to do it for themselves*
	Looking ahead	*Now I'm just doing the massage for lymphoedema and exercises only if I feel the problem*		
Being the 'perfect' therapist		*she asked me how I felt and it was very much about me and my progress*		*It almost like it made you be the perfect physio and the perfect way you should treat patients but you don't always have time to do that*
Delivering physiotherapy to women with breast cancer	Meeting the needs of women with breast cancer	*I stopped doing the exercises for three or four days if I was ill and after that it was more difficult to do the exercises after*		*they'd start their chemotherapy and then it was a whole different ball game because it was just kind of managing their fatigue and we struggled to get people back in for appointments*
	Emotional support for physiotherapists			*there were times where it was upsetting to hear*
	Physiotherapists' time, skills, and organisational integration			*I would say giving them the choice of exercise is time consuming which you wouldn't have in real life, you wouldn't have the time*<br><br>*There's not necessarily the integration with like the nurses or the lymphedema team, we are quite a separate team*<br><br>*Being able to advise people a bit more around like scar massage or kind of…any of the manual treatments that we could've done and when is right and wrong to use them* [was difficult]

For peer review only

For peer review only - http://bmjopen.bmj.com/site/about/guidelines.xhtml

Healing

This theme refers to trial participants' and physiotherapists' comments about how the exercise intervention shaped the experience of healing for the women with breast cancer.

Reassurance

In the acute period after surgery, participants from both the intervention and control groups reported feeling afraid to move their upper body. This is known as kinesiophobia [33, 34]. They felt unable to do the exercises prescribed in the Breast Cancer Care information leaflet.

> *It's quite tender…you don't feel like you ought to be doing it…you feel like it's too soon…I was aching so much that I just thought 'I just can't do this'.* – Qualitative Respondent (QR24) (Age 51, Control arm participant)

> *Because you don't know whether it's good or not, do you know what I mean, you don't know if you're doing well or not or if this is where you would be, you know, or you should be and it was quite nice because you got 'oh no you're doing really well' or 'oh yeah that will be tight' and it was that… it was quite nice to have the feedback* – QR12 (Age 55, Intervention arm participant)

Participants allocated to the intervention arm subsequently felt reassured by physiotherapists that they were capable and able to move. They felt reassured that bodily sensations, such as stiffness, were normal and not something to worry about. Physiotherapists felt that they were able to increase participants' confidence in moving their bodies, and that this lifted participants' confidence more broadly.

> *The, er, physiotherapist…was able to tell you whether you were doing things right or wrong or how things were going within your body.* – QR09 (Age 69, Intervention arm participant)

> Interviewer: What do you think they get out of coming to see you?
>
> PT02 (Physiotherapist): *I think confidence. Confidence to actually move…confidence to look after themselves, that they can do things*

Some described this as giving participants 'permission' to move, which was necessary to prevent movement restrictions in the upper body.

For peer review only - http://bmjopen.bmj.com/site/about/guidelines.xhtml

Making progress

This theme refers to physical improvements felt by participants in the intervention arm. This included how far they could stretch and how strong they were. Improvements were measurable and tangible, and participants highlighted the central role of the physiotherapist in creating this sense of progress.

> *You saw results and sometimes with your cancer…you don't see results until the end 'til they say 'You're all clear' you are just going through awful, awful, awful praying and hoping…But it is a really positive thing to think 'Oh* something *is getting better'.* – QR12 (Age 55, Intervention arm participant, participant's emphasis)

> *You could kind of measure it yourself and assess it yourself because you knew how far you could get your arm up…You could feel when, when things started to get a bit better.* – QR08 (Age 43, Intervention arm participant)

Where intervention participants spoke about the improvement they felt in the months following their surgery, control arm participants also spoke about improvement, but for them this remained an ongoing process even more than 12 months on.

> *When I'm washing myself and, and if I touch myself I'm tender but I suppose that will go* – QR17 (Age 67, Control arm participant)

> *I have still got a seroma on my chest which is a bit of a nuisance which is, um, sort of swelling of fluid isn't it. Um, it's less than it was and I think it's gradually going 'cause it was enormous at the very beginning but it's getting less* – QR16 (age 79, Control arm participant)

Over time, intervention participants progressed from gentle stretching to more advanced stretching and strengthening exercises as they improved. Graduating to harder exercises gave them a sense of achievement.

> *When we would do the exercises and when we would move the kind of categories in the folder that was given, that made me feel good and made me want to kind of continue.* – QR13 (Age 28, Intervention arm participant)

Progression was fulfilling and rewarding, particularly in the context of cancer treatment where a sense that they were improving or getting better was lacking. To be able to measurably perceive progress in strength and movement helped to restore a sense of bodily autonomy for the women who felt disempowered by cancer

For peer review only - http://bmjopen.bmj.com/site/about/guidelines.xhtml

treatment. During this profoundly difficult time of undergoing cancer treatment, feeling improvement and graduating to harder exercises helped them to feel that they *were* getting better, at least in some way.

Helping myself

During breast cancer treatment, women passively receive treatment [7-9, 35]. One participant described it as being "a professional waiter, you just sit and wait, and you just let everyone do what they're doing" - QR23 (Age 62, Control arm participant).

In collaboration with their physiotherapist, participants receiving the exercise intervention could choose which exercises they performed from a menu, selecting exercises they felt most confident and comfortable doing. The physiotherapists felt that joint-decision making was a patient-centred approach which added to trial participants' sense of ownership and control of their exercises. Physiotherapists and participants both noted that this gave participants an opportunity to be pro-active, taking control of one aspect of their recovery. This sense of responsibility and ownership motivated them to exercise.

> *I think it was more than the exercise. I think it was because you were doing something, because so much of um cancer care is being done to you…It was just quite nice to have something proactive for you to do rather than just turn up and have the drugs.* – QR12 (Age 55, Intervention arm participant*)*

> *I think when we were sort of promoting why we think the exercises were useful I talked about self-determination this is something that you can do for yourself and your care…particularly the way it was designed that enabled the patients to say well we could do this exercise or we could do that one.* – PT10 (Physiotherapist)

This was a key difference to the control arm interviewees. Most control arm participants spoke about accepting postoperative problems or just waiting for them to improve over time, apart from a few highly motivated individuals who described inventing their own exercises.

> *Lifting up now and I can feel the stretching down that left-hand side, but, um, you know I don't know, I suppose it's had trauma.* – QR15 (Age 44, Control arm participant)

> *The tightness on my chest does limit movement sometimes and it's sort of more of a discomfort than a pain and just a blessed nuisance really but everyone I've seen says it's normal that they take a while and*

For peer review only - http://bmjopen.bmj.com/site/about/guidelines.xhtml

> *it's nothing they can do so it will just go when it's ready I suppose and I kind of live with it* – QR17 (Age 67, Control arm participant)

Wanting to be a 'good patient' and doing as one was told motivated control group participants to follow the exercises on the Breast Cancer Care leaflet.

> *Well I'd like to think I was a good patient, I started my exercises the day after I came out of hospital.* – QR26 (Age 56, Control arm participant)

> *Just the fact that the hospital gave them you and, you know, they know what they're talking about. You do it because you've been told to.* – QR23 (Age 62, Control arm participant)

This is in contrast to the sense of self-determination, control, and progress described by participants receiving the intervention.

Looking ahead

Some participants continued to draw on knowledge gained from the intervention to alleviate ongoing problems with tightness and stiffness, and appeared to feel quite confident in managing this in the future.

> *Now I'm just doing the massage for lymphoedema and exercises only if I feel the problem…For example if, if I feel the problem to reach the shelf I'm taking* [the] *band and I might warm it up just do the exercises with the elastic band exactly for this movement.* – QR10 (Age 50, Intervention arm participant)

> *In a couple of months or so, I would like to kind of start using weights so that I can strengthen my arms…It's kind of like building up the strength that I was building towards whilst I was doing the* [PROSPER] *exercises before.* – QR13 (Age 28, Intervention arm participant)

They felt assured that continuing with such activities would help them, and that they would know what to do or where to seek help if required.

For peer review only - http://bmjopen.bmj.com/site/about/guidelines.xhtml

Being the 'perfect' therapist

This theme describes the physiotherapists' perspectives on the trial intervention compared to their usual practice and how it enabled them to deliver an optimal service.

> *It almost like it made you be the perfect physio and the perfect way you should treat patients but you don't always have time to do that.* – PT03 (Physiotherapist)

> *Agreed goals* [and] *agreed exercises actually that should be what we're doing anyway that shouldn't be anything radically different but sometimes because of time pressures you don't…If you work more collaboratively with patients there are massive benefits to it and I think it just reinforced that for me.* – PT10 (Physiotherapist)

This was supported by participant responses, where they described the relationship they built with their physiotherapist.

> *For me, you know, having the same… the same desired outcome as the physiotherapist and* [wife] *you know, kind of, being all, all, all wanting the same thing. And it kind of felt if I did those things then I would eventually achieve it.* – QR08 (Age 43, Intervention arm participant)

> *She's brilliant, she's so lovely and fantastic hugger that's what I found if somebody you meet is happy to give you a hug when you are in this kind of situation it… it just makes everything so much easier… you* [physiotherapists] *not only do you do your jobs but when you're dealing with people like me you are counsellors as well* – QR12 (Age 55, Intervention arm participant)

Physiotherapists felt that having longer appointment times and an emphasis on shared goals and shared decisions, both of which encouraged exercise adherence, represented an ideal way of working. Many of the therapists remarked that they were pleased to be able to offer this service to people with breast cancer because of their previous experience of treating women struggling with chronic immobility, pain, and psychological issues in the longer term as a result of shoulder problems following breast cancer surgery.

> *When you pick up those patients* [later] *they come with a lot of emotional baggage and sort of their belief systems and it may have been years since they used their shoulder normally and then you know again if you've got body dysmorphic issues and they've been carrying that around for two years that's a lot more challenging to support.* – PT06 (Physiotherapist)

For peer review only - http://bmjopen.bmj.com/site/about/guidelines.xhtml

We get people coming in about two years later and they've never touched their scar, they never saw a physio, they're stiff, their scar's horrible, they've got awful myofascial trigger points and tightness…They still think two years down the line they're going to hurt themselves if they over stretch so if you get them in at the early stage then it's just better…I had a lady who had a mastectomy it was three years later she never went back to work, she never went back to any exercise, she never touched her scar, her mental wellbeing was like absolutely awful when I first started seeing her because she just didn't even know that she could have her life back. – PT01 (Physiotherapist)

Physiotherapists connected this to the broader organisation of the NHS, and the need allocate resources to preventive care.

I think we work too much reactive in the NHS don't we and I think a direction to move in is work in prevention rather than cure. – PT02 (Physiotherapist)

Delivering physiotherapy to women with breast cancer

This theme reports views on delivering a new physiotherapist-led intervention for individuals with breast cancer.

Meeting the needs of women with breast cancer

Participants and physiotherapists suggested that adjuvant treatment, such as chemotherapy, interfered with the participants' ability to maintain the exercise programme. After stopping the exercises when they felt unwell, it was physically more difficult to start doing the exercises again. Physiotherapists reflected that intervening at this point may have helped encourage and motivate participants to continue.

On day 17 after chemotherapy it has been a struggle…the last three weeks with the first lot of chemo this…[doing the exercises has] *been a lot harder than I ever anticipated.* – QR11 (Age 49, Intervention arm participant)

A patient would come in for their first appointment and probably just post-surgery and most of them were quite positive had quite a lot of goals… they'd start their chemotherapy and then it was a whole

For peer review only - http://bmjopen.bmj.com/site/about/guidelines.xhtml

different ball game because it was just kind of managing their fatigue and we struggled to get people back in for appointments. – PT02 (Physiotherapist)

The physiotherapists noted that participants needed emotional support, and that it was difficult to provide this in a curtained cubicle in an open-plan space, where they potentially felt more vulnerable.

We're working in curtained cubicles a lot of the time and I felt that didn't set the tone, I think if you're asking someone to take their bra off . – PT10 (Physiotherapist)

Two therapists felt that physiotherapists should be female as they would better understand the meaning of losing a breast and more able to engage in the emotional and physical work of treating women with breast cancer.

They would probably connect better with a female and I was surprised how much women wanted to talk to me about their connection with their breasts so for a lot of them they felt like that was their femininity or that was um a connection to their womanhood and so I think most guys couldn't relate to how that feels so I could get where they were coming from. – PT08 (Physiotherapist)

This issue was not mentioned by the trial participants we interviewed.

Emotional support for physiotherapists

Physiotherapists typically provide emotional support to their patients, however, some therapists highlighted particular challenges in relation to this group due to the context of cancer treatment, for example, people with cancer were fearful of dying from breast cancer. This was in contrast to their usual caseload which often involved caring for people with chronic musculoskeletal conditions.

I am a person who cries quite easily so I was like 'Ok I need to keep things under control myself because I am the professional'… If I was to do it longer term I would need some better kind of guidance and help to deal with that…sometimes I felt a little bit lost. – PT08 (Physiotherapist)

We were lucky because we had each other but there were times where it was upsetting to hear…If we were permanent members of staff in oncology you would be given some…de-briefing or kind of decompression but we were never offered that… both of us have had very close relatives die because of cancer…nobody considered that at all. – PT06 (Physiotherapist)

For peer review only - http://bmjopen.bmj.com/site/about/guidelines.xhtml

The physiotherapists felt they would need emotional support if they worked routinely with people with breast cancer.

Physiotherapists' time, skills, and organisational integration

Delivering the intervention was time-consuming for physiotherapists.

> *I would say giving them the choice of exercise is time consuming which you wouldn't have in real life, you wouldn't have the time.* – PT09 (Physiotherapist)

Trial appointments were longer than usual and there were doubts about how this could be practically implemented as part of routine NHS clinical care given current time restrictions on appointments. The physiotherapists felt confident in identifying and treating physical shoulder problems, but often expressed a need for training about breast cancer, its treatments, and cancer specific complications. Cording, lymphedema, and seroma were unfamiliar postoperative complications to some physiotherapists until they took part in the trial.

> *We are MSK* [musculoskeletal] *physios and we know what a tight shoulder is and we know how to get it moving, so actually the assessment and the exercises wasn't so much of a worry, but patients occasionally asked me a question that maybe I couldn't answer…the background behind the cancer, a bit more about the actual surgical techniques they did and why and a little bit more about the reasoning of why lymphedema and cording does actually develop and what it means, I might have benefitted from more training from that aspect.* – PT03 (Physiotherapist)

> *Being able to advise people a bit more around like scar massage or kind of…any of the manual treatments that we could've done and when is right and wrong to use them* [was difficult]. – PT02 (Physiotherapist)

Physiotherapists felt disconnected from the surgical or oncology team treating the person with breast cancer which was challenging.

> *There's not necessarily the integration with like the nurses or the lymphedema team, we are quite a separate team from them so I think it does need to be a multi-disciplinary approach and because we're not involved with them it makes it a little bit difficult* [to know] *whether we should or shouldn't be doing those interventions.* – PT03 (Physiotherapist)

For peer review only

For peer review only - http://bmjopen.bmj.com/site/about/guidelines.xhtml

I sometimes found it difficult to ask about things like chemo, radiotherapy and repeat surgeries because I almost felt like it was something that I should know… that's what I'd want as a healthcare professional I want them to know what's going on I shouldn't have to tell you when I am having my chemo or this is happening. – PT05 (Physiotherapist)

Better integration with the oncology team would have given them greater understanding of the specific individual's treatment schedule as they sometimes felt uncertain about whether the interventions were appropriate at a particular stage of cancer treatment.

For peer review only

Discussion

This qualitative study embedded within a large multicentre clinical trial makes a unique contribution to the literature. Our study illustrates that an individual supported exercise intervention is perceived as acceptable and beneficial by both women with breast cancer and physiotherapists. Comparing the intervention and control arm enabled us to demonstrate that the intervention helped participants feel empowered and regaining a sense of control, whereas participants in the control arm spoke of passively accepting the upper limb limitations they experienced. Previous studies have explored perceptions of exercise in the context of an exercise intervention [36-38], but this is the first to include the perspectives of both intervention and control group participants, as well as physiotherapists delivering the intervention. We gained multiple perspectives on the same issue, and included all stakeholders in the study. This allowed us to triangulate and identify themes which were present across all groups. By using qualitative methods, we elicited the particular elements of the intervention which helped motivate participants, and those which were easier or more difficult to deliver in the clinical setting. This intervention is the first early structured physiotherapy-led home-based exercise intervention to be tested in women with breast cancer in the UK. An understanding of the acceptability of the intervention to women with breast cancer and physiotherapists will inform future implementation strategies if the intervention is clinically and cost-effective.

Uncertainty has been identified as a feature of the experience of cancer [39, 29]. The subtheme of 'making progress' showed how witnessing improvement for themselves in terms of strength and stretching stood out in sharp contrast with the uncertainty surrounding cancer and its treatment. Participants also gained a sense of control over their progress, through being involved in choosing exercises, and through taking responsibility for

completing their exercises each day (subthemes of 'helping myself' and looking ahead'). This combination appeared to restore participants' sense of autonomy over their bodies, and improved their wellbeing as they felt less disempowered and hopeless. This echoes previous research which found feelings of increased empowerment when people with breast cancer participated in physical activity during active treatment [40-42]. These experiences contrasted to those in the control group, who did not experience the same sense of empowerment and progress. Specific aspects of the intervention which contributed to this sense of control over and above usual care were the contact with physiotherapists and the reassurance this provided, the sense of progress working through the prescribed programme as exercises increased in difficulty, and the shared decision-making used to select the home exercises. Previous research has found that participating in a group activity can be a way of forgetting about the illness [36]. Our study illustrates this can also be true for home-based or individually supported exercise programmes.

Being diagnosed with a serious illness such as cancer can cause an individual to lose trust and confidence in their bodily knowledge and of what their bodies are capable of doing [12, 43-45]. The women in this study reported kinesiophobia (fear of movement) in the acute period following surgery, but those in the intervention arm felt the intervention helped them overcome this (subtheme of 'reassurance'). Kinesiophobia has been shown to be associated with lymphoedema and greater pain intensity [33, 34]. Physiotherapists were able to reassure women that their bodily sensations were normal, and gave them confidence to push themselves physically which motivated them to adhere to the programme. The interview data suggested that the role of the physiotherapist in affirming this progress and confidence was crucial. Physiotherapists provided invaluable emotional support, as participants unburdened onto them and shared their fears about the future and their bodies.

The physiotherapists enjoyed seeing positive improvements in the participants, and felt passionate about delivering what they viewed as high quality care to individuals with breast cancer (subtheme of 'being the perfect therapist'). Physiotherapists felt satisfaction in being able to take preventive action against problems arising in the future for these women. Intervention arm participants also appreciated the supportive nature of the intervention, sharing decisions and working together towards the same goal. Other authors have called for a more proactive model of health care provision for this patient group, and identified the need to improve physiotherapists' confidence in supporting those with breast cancer [46]. Our physiotherapists felt that they needed better integration with the rest of the individual's healthcare providers (final subtheme of 'Physiotherapists' time, skills, and organisational integration'). Other studies have also emphasised the importance of aligning expectations and knowledge about exercise based rehabilitation across the whole cancer care team [5] Challenges to the exercise

For peer review only - http://bmjopen.bmj.com/site/about/guidelines.xhtml

programme were the side effects of treatment, in particular fatigue, which has been highlighted in other research as a barrier to exercise for people with breast cancer [36]. If a physiotherapist can provide motivation and encouragement during chemotherapy, this may improve adherence to exercise. However, it is also important to ensure that physiotherapists are sympathetic to treatment-related issues, and can tailor programmes during these periods of fatigue [36].

The theme 'Delivering physiotherapy to women with breast cancer' highlighted considerations for the implementation of an exercise intervention for breast cancer patients. The intervention should be delivered in a private walled room, ideally with a specifically-trained (female) physiotherapist who is part of the multi-disciplinary oncology team caring for the person. The most important ingredient of the intervention was contact with the physiotherapists, suggesting resources should be focused on training and supporting physiotherapists to provide this care. Some physiotherapists reported feeling upset when treating patients because of the woman's distress or their own experiences of cancer. This suggests that healthcare professionals caring for oncology patients should be given the opportunity of debriefing and emotional support. This is an important consideration when designing future interventions for this group.

In the PROSPER trial, participants underwent a one hour assessment and then subsequent 30-minute follow-up appointments. Routine UK physiotherapy outpatient appointments are often 40 minutes (assessment) and 20 minutes (follow-up). Physiotherapists worked with participants to select the exercises. This may be challenging to deliver in a resource-stretched NHS context. However, longer appointments with physiotherapists, creating shared goals, and making shared decisions about exercises, were viewed as the most important ingredients in the successful delivery of the intervention. This was brought out in all our themes. Other studies have highlighted how autonomy of choice over exercises may increase motivation and adherence [37, 47]. Additionally, we provided the PROSPER materials in an attractive ring binder with colour photographs, laminated sheets, and provided exercise diary handouts. Trial participants said the diary was useful as a prompt to remember to do their exercises, and it was helpful to see photographs of the exercises.

Acting more proactively by providing good access to physiotherapy treatment early after, or alongside, breast cancer treatment could help to reduce the number of people with cancer (or a history of cancer) presenting with musculoskeletal complications [48]. Although our physiotherapist-participants felt very comfortable with aspects of the intervention such as improving shoulder mobility, they expressed a need for greater training, support, and guidance in relation to specific issues such as cording and lymphoedema. The physiotherapists delivering the

For peer review only - http://bmjopen.bmj.com/site/about/guidelines.xhtml

PROSPER intervention were musculoskeletal specialists with limited experience in treating individuals with breast cancer in the acute postoperative period. Physiotherapists in the UK receive little training in rehabilitation following cancer treatment, reflected by the limited centres across the UK with physiotherapists specialised in oncology [48]. Given the increasing number of people surviving cancer and living with the consequences of cancer treatment, there is an urgent need in the UK to upskill physiotherapists in cancer-related rehabilitation to allow people with breast cancer better access to this type of rehabilitation.

Conclusion

This study has highlighted how a physiotherapist-led home exercise programme, with built-in progression and shared decision-making, helped women undergoing breast cancer treatment gain a restored sense of control over their wellbeing, and empowered them during a highly disempowering experience.

Author statement

JB is Chief Investigator of the PROSPER Trial. JB/SR/EW/HR/BM contributed to the study protocol. SR collected and analysed the data. JB/BM/HR/JB/EW assisted with analysis and interpretation. SR led the writing of the paper, and all other authors contributed to writing and editing.

Funding

Funding for the PROSPER trial was provided by the Health Technology Assessment programme of the National Institute for Health Research (NIHR) (Project Number 13/84/10). JB is supported by NIHR Research Capability Funding via University Hospitals Coventry and Warwickshire. EW is supported by the NIHR Applied Research Collaboration Oxford and Thames Valley.

Conflicts of interest

The authors have no conflicts of interest.

For peer review only - http://bmjopen.bmj.com/site/about/guidelines.xhtml

For peer review only

Data availability statement

The datasets generated during and/or analysed during the current study are not publicly available due to the need to protect the identity of participants.

Patient and Public Involvement

Patients partnered with us for the design of the study, the informational material to support the qualitative research, and the burden of the interview from the perspective of women with breast cancer.

Acknowledgements

We would like to thank all the physiotherapists and trial participants who took part in this interview study. We dedicate this paper to Elizabeth (Lizzie) Abbey (nee Fort) who passed away on 3rd April 2018. Lizzie helped set up the trial and was a highly valued member of the PROSPER team.

References

1. McNeely ML, Campbell K, Ospina M, Rowe BH, Dabbs K, Klassen TP et al. Exercise interventions for upper-limb dysfunction due to breast cancer treatment. Cochrane Database Syst Rev. 2010(6). doi:10.1002/14651858.CD005211.pub2.
2. Mejdahl MK, Andersen KG, Gärtner R, Kroman N, Kehlet H. Persistent pain and sensory disturbances after treatment for breast cancer: six year nationwide follow-up study. BMJ. 2013;346:f1865.
3. Cardoso F, Kyriakides S, Ohno S, Penault-Llorca F, Poortmans P, Rubio IT et al. Early breast cancer: ESMO Clinical Practice Guidelines for diagnosis, treatment and follow-up. Ann Oncol. 2019. doi:10.1093/annonc/mdz173.
4. Excellence NIfC. Early and locally advanced breast cancer: diagnosis and management NICE guideline [NG101]. 2018. https://www.nice.org.uk/guidance/ng101/chapter/Recommendations. Accessed 31st January 2020.
5. Dennett AM, Harding KE, Reed MS. The challenge of timing: a qualitative study on clinician and patient perspectives about implementing exercise-based rehabilitation in an acute cancer treatment setting. Support Care Cancer. 2020. doi:10.1007/s00520-020-05436-7.
6. Fisher MI, Howell D. The power of empowerment: An ICF-based model to improve self-efficacy and upper extremity function of survivors of breast cancer. Rehabil Oncol. 2010;28(3).
7. Dunn J, Steginga SK. Young women's experience of breast cancer: defining young and identifying concerns. Psychooncology. 2000;9(2):137-46.

For peer review only - http://bmjopen.bmj.com/site/about/guidelines.xhtml

For peer review only

8. Thomas-MacLean R. Memories of treatment: the immediacy of breast cancer. Qual Health Res. 2004;14(5):628-43. doi:10.1177/1049732304263658.
9. Little M, Jordens CF, Paul K, Montgomery K, Philipson B. Liminality: a major category of the experience of cancer illness. Soc Sci Med. 1998;47(10):1485-94.
10. McCann L, Illingworth N, Wengström Y, Hubbard G, Kearney N. Transitional experiences of women with breast cancer within the first year following diagnosis. J Clin Nurs. 2010;19(13-14):1969-76.
11. Trusson D, Pilnick A, Roy S. A new normal?: Women's experiences of biographical disruption and liminality following treatment for early stage breast cancer. Soc Sci Med. 2016.
12. Rees S. No one scans you and says 'you're alright now': the experience of embodied risk for young women living with a history of breast cancer. Health, Risk & Society. 2018:1-13. doi:10.1080/13698575.2018.1539468.
13. Kim S, Han J, Lee MY, Jang MK. The experience of cancer-related fatigue, exercise and exercise adherence among women breast cancer survivors: Insights from focus group interviews. J Clin Nurs. 2020;29(5-6):758-69. doi:10.1111/jocn.15114.
14. Livsey L, Lewis K. Breast cancer survivors' perceptions of participating in a supervised exercise intervention: An exploratory review of the literature. Women Health. 2018;58(9):1017-36. doi:10.1080/03630242.2017.1372844.
15. Kenyon K Mres PT, Hebron C PhD PT, Vuoskoski P PhD PT, McCrum C Dprof PT. Physiotherapists' experiences of managing upper limb movement impairments due to breast cancer treatment. Physiother Theory Pract. 2018:1-14. doi:10.1080/09593985.2018.1480077.
16. Bruce J, Williamson E, Lait C, Richmond H, Betteley L, Lall R et al. Randomised controlled trial of exercise to prevent shoulder problems in women undergoing breast cancer treatment: study protocol for the prevention of shoulder problems trial (UK PROSPER). BMJ Open. 2018;8(3):e019078. doi:10.1136/bmjopen-2017-019078.
17. Richmond H, Lait C, Srikesavan C, Williamson E, Moser J, Newman M et al. Development of an exercise intervention for the prevention of musculoskeletal shoulder problems after breast cancer treatment: the prevention of shoulder problems trial (UK PROSPER). BMC Health Serv Res. 2018;18(1):463. doi:10.1186/s12913-018-3280-x.
18. Archer MS. Realist social theory: The morphogenetic approach. Cambridge University Press; 1995.
19. Bhaskar R. A realist theory of science. Routledge; 2013.
20. Braun V, Clarke V. Reflecting on reflexive thematic analysis. Qualitative Research in Sport, Exercise and Health. 2019;11(4):589-97. doi:10.1080/2159676X.2019.1628806.
21. O'Brien BC, Harris IB, Beckman TJ, Reed DA, Cook DAJAM. Standards for reporting qualitative research: a synthesis of recommendations. 2014;89(9):1245-51.
22. Ltd QIP. NVivo qualitative data analysis software. QSR International Pty Ltd. Stokholm, Sweden; 2008.
23. Guest G, MacQueen KM, Namey EE. Applied thematic analysis. Sage; 2011.
24. Boyatzis RE. Transforming qualitative information: Thematic analysis and code development. sage; 1998.
25. Charmaz K. Constructing grounded theory. Sage; 2014.
26. Braun V, Clarke V. To saturate or not to saturate? Questioning data saturation as a useful concept for thematic analysis and sample-size rationales. Qualitative Research in Sport, Exercise and Health. 2019:1-16. doi:10.1080/2159676X.2019.1704846.
27. Hennink MM, Kaiser BN, Marconi VC. Code saturation versus meaning saturation: how many interviews are enough? Qual Health Res. 2017;27(4):591-608.
28. Rees S. A qualitative exploration of the meaning of the term "survivor" to young women living with a history of breast cancer. European journal of cancer care. 2018;27(3):e12847.
29. Rees S. 'Am I really gonna go sixty years without getting cancer again?'Uncertainty and liminality in young women's accounts of living with a history of breast cancer. Health:. 2017;21(3):241–58. doi:10.1177/1363459316677628.
30. Rees S, Young A. The experiences and perceptions of women diagnosed with breast cancer during pregnancy. Asia-Pacific journal of oncology nursing. 2016;3(3):252-8. doi:10.4103/2347-5625.189814.
31. Hutchinson A, Rees S, Young A, Maraveyas A, Date K, Johnson MJ. Oral anticoagulation is preferable to injected, but only if it is safe and effective: An interview study of patient and carer experience of oral and injected anticoagulant therapy for cancer-associated thrombosis in the select-d trial. Palliat Med. 2019;33(5):510-7.
32. Lincoln YS, Guba EG. But is it rigorous? Trustworthiness and authenticity in naturalistic evaluation. New directions for program evaluation. 1986;1986(30):73-84.
33. Gencay Can A, Can SS, Ekşioğlu E, Çakcı FA. Is kinesiophobia associated with lymphedema, upper extremity function, and psychological morbidity in breast cancer survivors? Turkish journal of physical medicine and rehabilitation. 2018;65(2):139-46. doi:10.5606/tftrd.2019.2585.

For peer review only - http://bmjopen.bmj.com/site/about/guidelines.xhtml

34. Martinez-Calderon J, Struyf F, Meeus M, Luque-Suarez A. The association between pain beliefs and pain intensity and/or disability in people with shoulder pain: A systematic review. Musculoskelet Sci Pract. 2018;37:29-57. doi:10.1016/j.msksp.2018.06.010.
35. Trusson D. Living with a new normal: women's experiences following treatment for early-stage breast cancer or DCIS: University of Nottingham; 2013.
36. Browall M, Mijwel S, Rundqvist H, Wengstrom Y. Physical Activity During and After Adjuvant Treatment for Breast Cancer: An Integrative Review of Women's Experiences. Integr Cancer Ther. 2018;17(1):16-30. doi:10.1177/1534735416683807.
37. Ingram C, Wessel J, Courneya KS. Women's perceptions of home-based exercise performed during adjuvant chemotherapy for breast cancer. Eur J Oncol Nurs. 2010;14(3):238-43. doi:10.1016/j.ejon.2010.01.027.
38. Luoma ML, Hakamies-Blomqvist L, Blomqvist C, Nikander R, Gustavsson-Lilius M, Saarto T. Experiences of breast cancer survivors participating in a tailored exercise intervention -a qualitative study. Anticancer Res. 2014;34(3):1193-9.
39. Halliday LE, Boughton MA, Kerridge I. Mothering and self-othering: The impact of uncertain reproductive capability in young women after hematological malignancy. Health Care Women Int. 2014;35(3):249-65.
40. Husebo AM, Karlsen B, Allan H, Soreide JA, Bru E. Factors perceived to influence exercise adherence in women with breast cancer participating in an exercise programme during adjuvant chemotherapy: a focus group study. J Clin Nurs. 2015;24(3-4):500-10. doi:10.1111/jocn.12633.
41. Crane-Okada R, Kiger H, Anderson NL, Carroll-Johnson RM, Sugerman F, Shapiro SL et al. Participant perceptions of a mindful movement program for older women with breast cancer: focus group results. Cancer Nurs. 2012;35(3):E1-10. doi:10.1097/NCC.0b013e31822539c5.
42. Bulmer SM, Howell J, Ackerman L, Fedric R. Women's perceived benefits of exercise during and after breast cancer treatment. Women Health. 2012;52(8):771-87. doi:10.1080/03630242.2012.725707.
43. Williams SJ. The vicissitudes of embodiment across the chronic illness trajectory. Body & Society. 1996;2(2):23-47.
44. Lindwall L, Bergbom I. The altered body after breast cancer surgery. International Journal of Qualitative Studies on Health and Well-being. 2009;4(4):280-7.
45. Crouch M, McKenzie H. Social realities of loss and suffering following mastectomy. Health:. 2000;4(2):196-215.
46. Levangie PK, Santasier AM, Stout NL, Pfalzer L. A qualitative assessment of upper quarter dysfunction reported by physical therapists treated for breast cancer or treating breast cancer sequelae. Support Care Cancer. 2011;19(9):1367-78. doi:10.1007/s00520-010-0959-x.
47. Jones LW, Courneya KS, Fairey AS, Mackey JR. Does the theory of planned behavior mediate the effects of an oncologist's recommendation to exercise in newly diagnosed breast cancer survivors? Results from a randomized controlled trial. Health Psychol. 2005;24(2):189.
48. Robinson M, Ward L, Mehanna H, Paleri V, Winter SC. Provision of physiotherapy rehabilitation following neck dissection in the UK. The Journal of Laryngology & Otology. 2018;132(7):624-7. doi:10.1017/S0022215118000671.

For peer review only - http://bmjopen.bmj.com/site/about/guidelines.xhtml

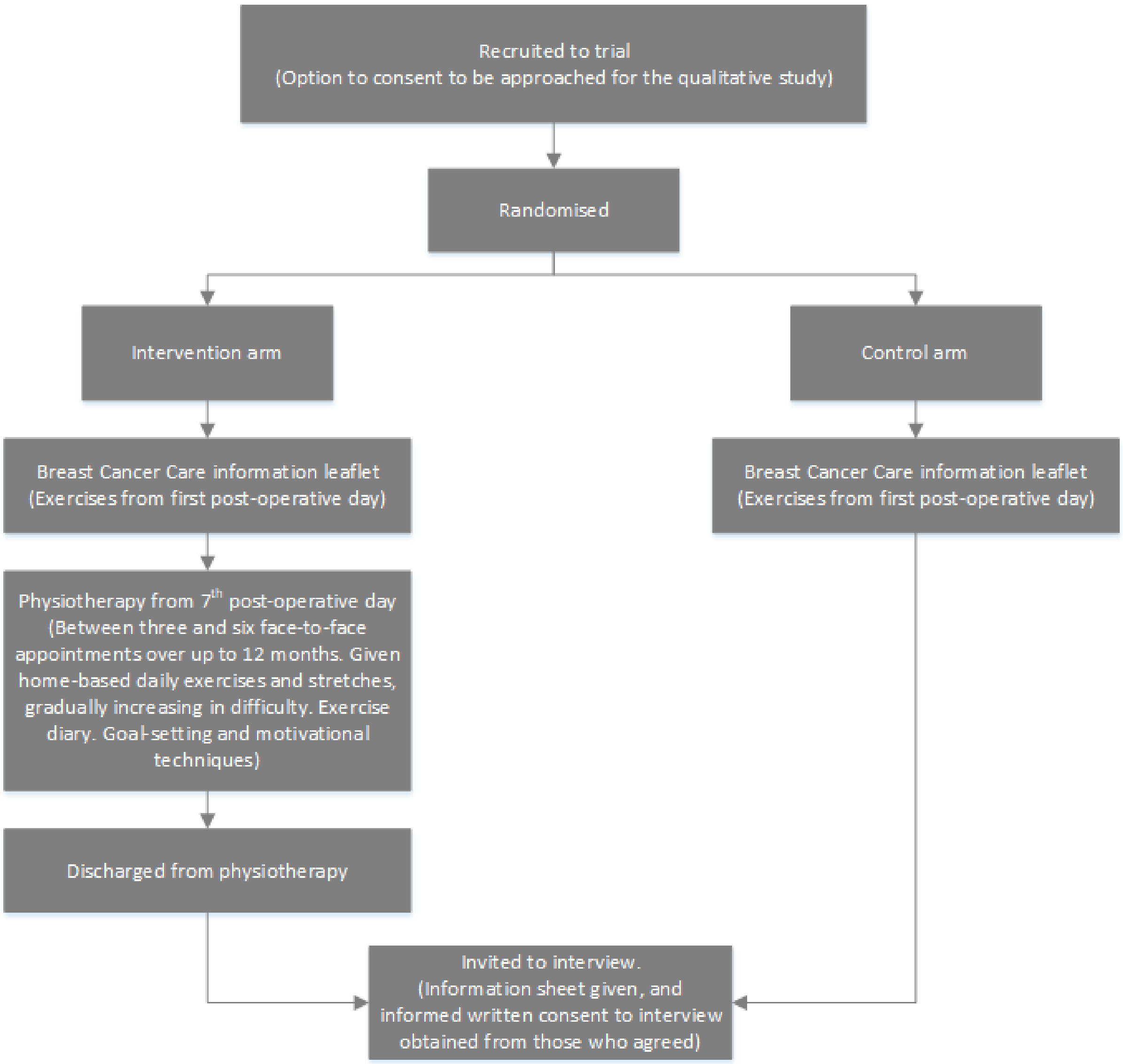
Recruited to trial
(Option to consent to be approached for the qualitative study)
Randomised
Intervention arm
Control arm
Breast Cancer Care information leaflet
(Exercises from first post-operative day)
Breast Cancer Care information leaflet
(Exercises from first post-operative day)
Physiotherapy from 7th post-operative day (Between three and six face-to-face appointments over up to 12 months. Given home-based daily exercises and stretches, gradually increasing in difficulty. Exercise diary. Goal-setting and motivational techniques)
Discharged from physiotherapy
Invited to interview. (Information sheet given, and informed written consent to interview obtained from those who agreed)

Standards for Reporting Qualitative Research (SRQR)*

http://www.equator-network.org/reporting-guidelines/srqr/

Title and abstract

	Page/line no(s).
**Title** - Concise description of the nature and topic of the study Identifying the study as qualitative or indicating the approach (e.g., ethnography, grounded theory) or data collection methods (e.g., interview, focus group) is recommended	1
**Abstract** - Summary of key elements of the study using the abstract format of the intended publication; typically includes background, purpose, methods, results, and conclusions	1-2

Introduction

**Problem formulation** - Description and significance of the problem/phenomenon studied; review of relevant theory and empirical work; problem statement	3-4
**Purpose or research question** - Purpose of the study and specific objectives or questions	4

Methods

**Qualitative approach and research paradigm** - Qualitative approach (e.g., ethnography, grounded theory, case study, phenomenology, narrative research) and guiding theory if appropriate; identifying the research paradigm (e.g., postpositivist, constructivist/ interpretivist) is also recommended; rationale**	5
**Researcher characteristics and reflexivity** - Researchers' characteristics that may influence the research, including personal attributes, qualifications/experience, relationship with participants, assumptions, and/or presuppositions; potential or actual interaction between researchers' characteristics and the research questions, approach, methods, results, and/or transferability	6-7
**Context** - Setting/site and salient contextual factors; rationale**	5
**Sampling strategy** - How and why research participants, documents, or events were selected; criteria for deciding when no further sampling was necessary (e.g., sampling saturation); rationale**	5
**Ethical issues pertaining to human subjects** - Documentation of approval by an appropriate ethics review board and participant consent, or explanation for lack thereof; other confidentiality and data security issues	6
**Data collection methods** - Types of data collected; details of data collection procedures including (as appropriate) start and stop dates of data collection and analysis, iterative process, triangulation of sources/methods, and modification of procedures in response to evolving study findings; rationale**	6

For peer review only

For peer review only - http://bmjopen.bmj.com/site/about/guidelines.xhtml

**Data collection instruments and technologies** - Description of instruments (e.g., interview guides, questionnaires) and devices (e.g., audio recorders) used for data collection; if/how the instrument(s) changed over the course of the study	6
**Units of study** - Number and relevant characteristics of participants, documents, or events included in the study; level of participation (could be reported in results)	7-8
**Data processing** - Methods for processing data prior to and during analysis, including transcription, data entry, data management and security, verification of data integrity, data coding, and anonymization/de-identification of excerpts	6
**Data analysis** - Process by which inferences, themes, etc., were identified and developed, including the researchers involved in data analysis; usually references a specific paradigm or approach; rationale**	6
**Techniques to enhance trustworthiness** - Techniques to enhance trustworthiness and credibility of data analysis (e.g., member checking, audit trail, triangulation); rationale**	6-7

Results/findings

**Synthesis and interpretation** - Main findings (e.g., interpretations, inferences, and themes); might include development of a theory or model, or integration with prior research or theory	9
**Links to empirical data** - Evidence (e.g., quotes, field notes, text excerpts, photographs) to substantiate analytic findings	10-18

Discussion

**Integration with prior work, implications, transferability, and contribution(s) to the field** - Short summary of main findings; explanation of how findings and conclusions connect to, support, elaborate on, or challenge conclusions of earlier scholarship; discussion of scope of application/generalizability; identification of unique contribution(s) to scholarship in a discipline or field	18-20
**Limitations** - Trustworthiness and limitations of findings	3

Other

**Conflicts of interest** - Potential sources of influence or perceived influence on study conduct and conclusions; how these were managed	6-7, 21
**Funding** - Sources of funding and other support; role of funders in data collection, interpretation, and reporting	21

*The authors created the SRQR by searching the literature to identify guidelines, reporting standards, and critical appraisal criteria for qualitative research; reviewing the reference lists of retrieved sources; and contacting experts to gain feedback. The SRQR aims to improve the transparency of all aspects of qualitative research by providing clear standards for reporting qualitative research.

For peer review only

For peer review only - http://bmjopen.bmj.com/site/about/guidelines.xhtml

**The rationale should briefly discuss the justification for choosing that theory, approach, method, or technique rather than other options available, the assumptions and limitations implicit in those choices, and how those choices influence study conclusions and transferability. As appropriate, the rationale for several items might be discussed together.

Reference:

O'Brien BC, Harris IB, Beckman TJ, Reed DA, Cook DA. **Standards for reporting qualitative research: a synthesis of recommendations.** *Academic Medicine*, Vol. 89, No. 9 / Sept 2014 DOI: 10.1097/ACM.0000000000000388

For peer review only

For peer review only - http://bmjopen.bmj.com/site/about/guidelines.xhtml
